# Supplementary material for: Segment anything in medical images
Source: Nat Commun. 2024 Jan 22;15:654. doi: 10.1038/s41467-024-44824-z (PMC10803759; doi:10.1038/s41467-024-44824-z)
Supplement: Supplementary file 1 — Supplementary Information [file 41467_2024_44824_MOESM1_ESM.pdf]

Supplementary

TABLE 1
Computed Tomography (CT) image datasets. Datasets marked with ∗ denote external validation sets and the remaining datasets are used for internal validation.

Dataset Name	Modality	Segmentation Targets	# of scans
AbdomenCT-1K [1], [2]	CT	Liver, kidneys, pancreas, spleen	1056
Adrenal-ACC-Ki67* [3]–[5]	CT	Adrenocortical carcinoma	53
AMOS-CT [6]	CT	Abdominal organ	200
AutoPET [7]	PET-CT	Whole-body tumor	900
COVID-19 Seg. Challenge [8], [9]	CT	COVID-19 infections	199
COVID-19-CT-Seg [10]	CT	COVID-19 infections, left lung, and right lung	20
GLIS-RT [11]	CT	Head tumor	75
HCC-TACE-Seg* [5], [12]	CT	Liver cancer	70
HECKTOR [13]	PET-CT	Head and neck tumor	524
INSTANCE [14]	CT	Hematoma	100
KiPA [15], [16]	CT	Kidney, tumor, renal artery, renal vein	70
KiTS [17]	CT	Kidney, tumor, cyst	489
LNQ2023 * [18]	CT	Mediastinal lymph node	393
Lymph Nodes [19], [20]	CT	Lymph nodes	176
MSD-Colon Tumor [21]	CT	Colon tumor	126
MSD-Hepatic Tumor [21]	CT	Hepatic tumor	303
MSD-Lung Tumor [21]	CT	Lung tumor	96
MSD-Pancreas [21]	CT	Pancreas, pancreas tumor	281
MSD-Spleen [21]	CT	Spleen	61
NSCLC Pleural Effusion [5], [22], [23]	CT	Pleural effusion	78
NSCLC Radiogenomics [24]	CT	Lung Tumor	88
ORG [25]	CT	Whole-body organs	140
SegTHOR [26]	CT	Esophagus, heart, aorta, trachea	40
StructSeg* [27]	CT	Nasopharyngeal cancer and lung cancer, with OAR and GTV	50
TotalSegmentator [28]	CT	Whole body organs	1204
WORD* [29]	CT	Abdominal organs	150

TABLE 2
Magnetic Resonance (MR) image datasets. Datasets marked with $*$ denote external validation sets and the remaining datasets are used for internal validation.

Dataset Name	Modality	Segmentation Targets	# of scans
ACDC* [30]	MR	Heart anatomies	150
AMOS-MR [6]	MR	Abdominal organ	40
ATLAS R2.0 [31]	MR-T1	Brain stroke	1271
Brain Tumor Dataset Figshare [32], [33]	MR-T1ce	Brain tumor	233
Brain TR-GammakKnife [34]	MR	Brain lesion	47
BraTS [35]–[39]	MR-T1, MR-T1CE, MR-T2, MR-FLAIR	Brain tumor	1251
CC-Tumor Heterogeneity* [40]	MR	Cervical cancer	7
CHAOS* [41]	MR-T1, MR-T2	Liver, kidney, spleen	60
crossMoDA [42]	MR	Brain tumor	227
FeTA [42]	MR-Fetal	Brain tissues	160
HaN-Seg* [43]	MR	Head organs	42
ISLES [44]	MR-DWI, MR-ADC, MR-FLAIR	Ischemic stroke lesion	180
I2CVB [45]	MR-T2, MR-DWI	Prostate	19
Meningioma-SEG-CLASS [46]	MR-T1ce, T2-FLAIR	Tumor (meningioma)	191
MMs [47]	MR	Heart anatomies	150
MSD-Heart [48]	MR	Left atrial	30
MSD-Prostate [21]	MR-ADC, MR-T2	Prostate	48
NCI-ISBI [49]	MR-ADC, MR-T2	Prostate	48
PI-CAI [50]	MR-bp	Prostate cancer	1584
PPMI [51]	MR-T1	Brain regions of Parkinson patients	1130
PROMISE [52]	MR-T2	Prostate	50
Qin-Prostate-Repeatability [21], [53]	MR	Left atrium	30
QUBIQ* [54]	MR	Prostate	52
Spine [55]	MR	Vertebrae	172
WMH [56]	MR-T1, MR-FLAIR	White matter hyper-intensities	60

TABLE 3
Chest X-Ray (CXR), Mammography, Optical Coherence Tomography (OCT), and Ultrasound image datasets. Datasets marked with $*$ denote external validation sets and the remaining datasets are used for internal validation.

Dataset Name	Modality	Segmentation Targets	# of images
Chest Xray Masks and Labels [57] [58]	CXR	Lung	704
Chest X-Ray (Pneumothorax) [59] [60]	CXR	Pneumothorax	2668
COVID-19 Radiography* [61] [62]	CXR	Lung, COVID-19 infection, lung opacity, viral pneumonia	21165
COVID-QU-Ex [63] [61] [64] [62] [65]	CXR	Lung, COVID infection	5826
JSRT [66]	CXR	Lung, heart	307
Lung [67] [68] [69]	CXR	Lung	30330
QaTa-COV19 [70]	CXR	COVID-19 infection	9258
CDD-CESM [5] [71] [72]	Mammography	Abnormal findings in breast tissue	1233
Intraretinal Cystoid Fluid [73] [74]	OCT	Cystoid macular edema	1460
OCT Images (DME) [75]	OCT	Diabetic macular edema	610
AbdomenUS [76]	Ultrasound	Gallbladder, kidney, liver, spleen, vessel	60
Breast Cancer [77]	Ultrasound	Benign & malignant breast lesion	647
CAMUS [78]	Ultrasound	Left ventricle endocardium, left atrium	21232
CT2USforKidneySeg [79]	Ultrasound	Kidney	4586
FH-PS-AOP [80]	Ultrasound	Pubic symphysis, fetal head	4000
HC [81]*	Ultrasound	Fetal head circumference	999
TN-SCUI [82]	Ultrasound	Benign & malignant thyroid nodules	3644
Nerve [83]	Ultrasound	Brachial plexus	5635

TABLE 4
Dermoscopy, Endoscopy, Fundus, and Pathology image datasets. Datasets marked with ∗ denote external validation sets and the remaining datasets are used for internal validation.

Dataset Name	Modality	Segmentation Targets	# of images
ISIC [84] [85] [86]	Dermoscopy	Skin cancer	3694
UWaterloo Skin Cancer* [87]	Dermoscopy	Skin cancer	206
BKAI-IGH NeoPolyp [88]	Endoscopy	Polyp	1000
CholecSeg8k [89] [90]	Endoscopy	Surgical tools and abdominal tissues	8080
Kvasir* [91]	Endoscopy	Polyp	1000
m2caiSeg [92]	Endoscopy	Surgical tools and abdominal tissues	307
PolypGen [93] [94] [95]	Endoscopy	Polyp	1412
RoboTool [96]	Endoscopy	Surgical tools	500
sisvse [97]	Endoscopy	Surgical tools and abdominal tissues	18218
IDRiD* [98]	Fundus	Optic disc	81
PAPILA* [99]	Fundus	Optic disc and cup	488
REFUGE [100] [101]	Fundus	Optic disc and cup	1200
GlaS@MICCAI2015* [102] [103]	Pathology	Adenocarcinomas	165
HuBMAP HPA [104]	Pathology	Function tissue unit (FTU) of large intestine	58
HuBMAP Hacking the Kidney [105]	Pathology	Glomeruli FTU	8

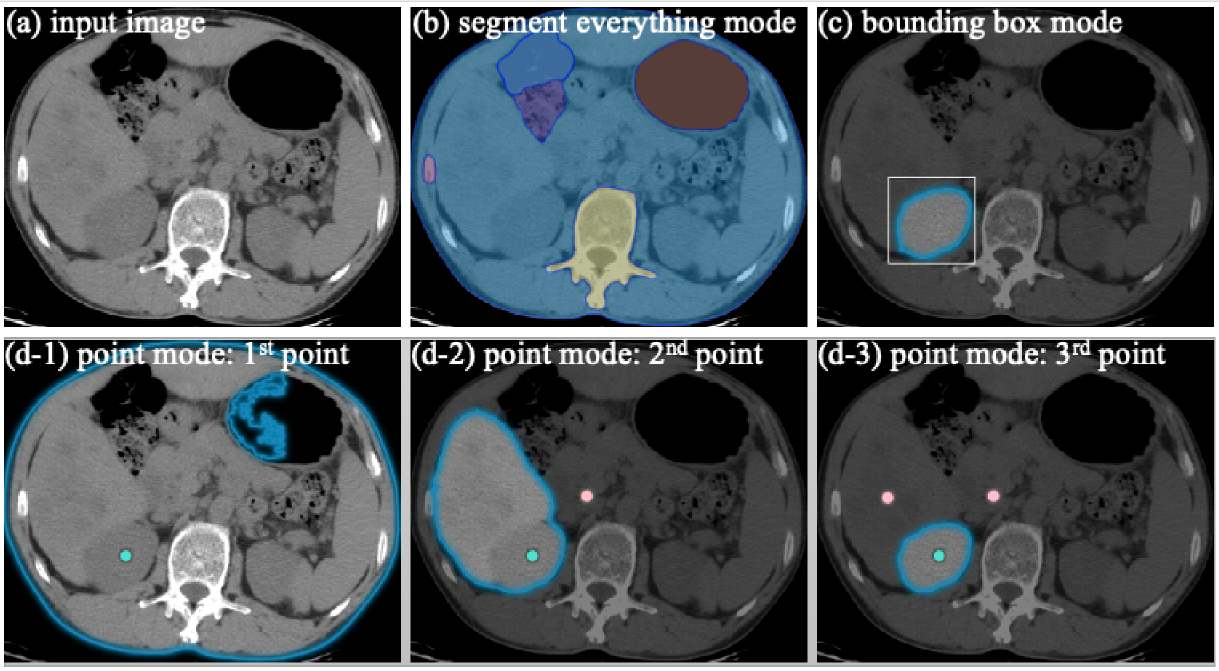

Fig. 1. Segmentation results of SAM based on different segmentation modes. The segment-everything mode divides the whole image into six regions based on the image intensity (b). However, such segmentation results have limited use because of two main reasons. On the one hand, the segmentation results do not have semantic labels. On the other hand, clinicians mainly focus on meaningful ROIs in clinical scenarios, e.g., the liver, kidneys, spleen, and lesions. The bounding box-based segmentation mode generates good results for the right kidney by just giving the upper-left and bottom-right points (c). For the point-based segmentation mode (d), we first give one foreground point to the center of the right kidney but the segmentation results include the whole abdomen tissues. Then, we add a background point on the over-segmentation regions. The segmentation mask shrinks to the liver and right kidney. After adding another background point on the liver, we finally obtain the expected kidney segmentation.

TABLE 5
The number of training images for modality-wise specialist models.

Modality	Num. of Training Images
Computed Tomography	362,229
Magnetic Resonance Imaging	442,818
X-Ray	36915
Mammography	986
Optical Coherence Tomography	642
Ultrasound	74761
Dermoscopy	2955
Endoscopy	194805
Fundus	960
Pathology	6239

TABLE 6
Internal validation results of SAM, specialist U-Net, specialist DeepLabV3+, and MedSAM on CT image segmentation tasks in terms of DSC and NSD. Scores are displayed as Median values (First quartile, Third quartile). The Wilcoxon signed-rank test was performed for the validation results of each target. Scores marked with $*$ indicate statistically significant superiority over SAM, while those marked with † denote statistically significant superiority of MedSAM over the specialist models (p-value $<$ 0.05). Source data are provided as a Source Data file.

Target	Modality	DSC (%)				NSD (%)			
		SAM	U-Net	DeepLabV3+	MedSAM	SAM	U-Net	DeepLabV3+	MedSAM
Aorta	CT	81.2 (74.3, 86.6)	92.6 (90.2, 96.2)*	90.1 (86.8, 92.3)*†	93.6 (92.4, 94.9)*	66.6 (57.6, 76.9)	92.2 (88.1, 97.4)*†	91.9 (85.9, 96.3)*†	98.2 (96.3, 99.1)*
COVID-19 Infection	CT	56.2 (50.6, 63.1)	82.3 (76.5, 84.2)*†	75.8 (72.9, 82.1)*†	87.9 (86.6, 91.9)*	64.8 (59.9, 74.1)	87.2 (85.6, 91.0)*†	85.6 (74.8, 89.6)*†	94.7 (90.5, 97.4)*
Colon Cancer	CT	69.3 (65.2, 77.9)	85.3 (84.0, 89.1)*	84.0 (80.8, 88.2)*	85.4 (83.2, 88.5)*	74.0 (70.9, 82.5)	92.5 (92.2, 98.2)*	89.4 (86.9, 97.1)*	92.1 (91.7, 97.8)*
Esophagus	CT	38.5 (40.2, 53.5)	70.1 (71.3, 74.6)*	58.0 (61.0, 71.8)	64.0 (64.5, 74.8)*	44.8 (45.4, 59.3)	70.0 (70.3, 74.9)*	68.0 (72.0, 73.5)	63.9 (66.6, 74.5)
Esophagus	CT	61.6 (53.0, 66.1)	83.0 (79.3, 87.1)*†	82.9 (80.1, 85.8)*†	85.4 (83.2, 88.4)*	57.2 (51.2, 65.1)	90.6 (84.3, 95.6)*†	91.7 (88.7, 95.2)*†	95.6 (93.1, 97.6)*
Gallbladder	CT	74.2 (60.1, 83.6)	89.4 (84.7, 94.0)*†	85.8 (81.2, 90.3)*†	91.5 (87.7, 94.4)*	69.2 (51.7, 80.5)	92.2 (84.7, 97.2)*†	88.2 (80.9, 93.5)*†	96.6 (92.8, 98.8)*
Glioblastoma	CT	65.3 (48.0, 73.2)	87.3 (86.5, 89.2)*†	85.6 (83.9, 87.4)*†	95.7 (95.3, 96.2)*	55.8 (52.9, 60.3)	79.2 (76.2, 85.1)*†	76.4 (73.1, 83.9)*†	96.7 (96.1, 98.4)*
Head-Neck Cancer	CT	40.2 (28.3, 46.9)	77.8 (75.9, 81.4)*†	77.9 (75.4, 80.9)*†	83.1 (79.5, 86.9)*	44.8 (38.8, 51.3)	74.3 (67.6, 79.8)*†	76.7 (72.0, 82.7)*†	83.3 (76.8, 88.3)*
Heart	CT	90.7 (91.0, 94.8)	92.7 (92.2, 93.2)*†	88.4 (88.8, 91.5)†	95.0 (95.0, 96.6)*	75.5 (77.8, 86.1)	80.5 (79.2, 82.9)*†	64.6 (69.1, 81.7)†	89.5 (89.9, 93.8)*
Inferior Vena Cava	CT	69.7 (63.2, 75.6)	86.3 (79.5, 90.4)*†	84.7 (80.8, 87.7)*†	89.8 (87.6, 92.0)*	54.8 (46.1, 61.3)	81.3 (66.1, 88.8)*†	80.5 (75.4, 85.9)*†	92.6 (89.0, 95.8)*
Intracranial Hemorrhage	CT	87.7 (87.4, 92.9)	93.2 (93.0, 95.9)	89.6 (89.2, 92.4)	95.2 (95.1, 96.7)	92.3 (92.1, 99.0)	99.4 (99.2, 99.7)*	97.6 (97.4, 99.3)	99.6 (99.6, 99.8)*
Kidney Cancer	CT	80.3 (68.8, 88.9)	92.0 (90.5, 94.1)*†	90.7 (87.8, 92.4)*†	95.0 (92.2, 96.7)*	72.3 (67.1, 78.7)	92.1 (87.8, 94.2)*†	88.5 (86.2, 93.1)*†	97.7 (96.2, 98.9)*
Left Adrenal Gland	CT	39.6 (32.8, 46.9)	77.1 (72.9, 83.0)*	68.9 (62.8, 73.7)*†	79.2 (75.1, 83.7)*	48.3 (38.2, 57.8)	88.4 (83.0, 92.5)*†	82.1 (76.3, 89.6)*†	93.5 (89.9, 96.8)*
Left Kidney	CT	89.6 (81.6, 94.3)	97.0 (94.2, 97.9)*†	94.1 (92.3, 95.2)*†	95.3 (94.2, 97.1)*	81.5 (74.7, 89.6)	95.9 (92.8, 98.8)*†	94.2 (91.4, 96.5)*†	97.8 (94.6, 99.0)*
Left Lung	CT	97.4 (96.2, 97.8)	98.7 (98.4, 98.8)*†	97.4 (97.1, 97.8)*†	98.3 (98.0, 98.5)*	86.3 (79.5, 91.2)	97.5 (96.6, 98.1)*†	92.8 (90.9, 94.6)*†	96.2 (95.5, 97.2)*
Liver	CT	87.1 (79.8, 91.6)	97.6 (96.9, 98.4)*†	95.5 (94.3, 96.4)*†	97.2 (96.7, 97.7)*	57.2 (48.2, 67.6)	96.1 (91.5, 98.3)*	87.8 (82.3, 93.1)*†	95.5 (91.9, 97.8)*
Liver Cancer	CT	68.5 (60.3, 80.8)	87.7 (82.4, 91.7)*	87.3 (83.4, 91.8)*	89.4 (83.4, 92.7)*	63.7 (56.2, 79.6)	88.3 (81.9, 92.6)*†	89.8 (82.2, 94.5)*†	92.7 (87.5, 95.9)*
Lung Cancer	CT	67.2 (56.0, 73.6)	88.0 (84.0, 89.7)*†	85.4 (81.3, 88.7)*†	93.8 (88.7, 95.1)*	62.7 (54.8, 71.8)	87.3 (84.0, 89.7)*†	83.7 (77.9, 87.6)*†	96.6 (92.7, 97.8)*
Lymph Nodes	CT	59.2 (55.2, 67.4)	80.3 (79.4, 83.7)*	79.1 (78.2, 82.6)*†	85.0 (84.1, 86.6)*	53.6 (51.2, 60.0)	84.2 (82.6, 91.3)*†	83.6 (81.0, 87.7)*†	91.3 (90.4, 94.8)*
Melanoma	CT	55.6 (50.9, 63.4)	74.2 (71.1, 80.5)*	73.0 (67.7, 78.2)*†	77.5 (73.1, 81.7)*	48.0 (44.6, 56.8)	66.5 (62.0, 71.5)*	61.8 (59.4, 69.4)*†	69.1 (65.3, 75.2)*
Pancreas	CT	58.0 (49.5, 70.0)	85.7 (82.0, 89.3)*	78.5 (71.7, 83.0)*†	85.5 (81.4, 89.1)*	54.6 (45.7, 66.2)	87.4 (78.9, 94.8)*	76.5 (69.3, 85.9)*†	87.4 (80.8, 93.9)*
Pancreas Cancer	CT	52.6 (48.5, 61.5)	75.8 (70.4, 81.9)*	77.4 (71.3, 85.1)*	77.8 (74.5, 83.9)*	52.9 (48.4, 63.0)	81.8 (75.9, 87.8)*	84.6 (79.6, 89.8)*	84.5 (76.3, 90.5)*
Pleural Effusion	CT	13.2 (13.2, 35.7)	82.5 (81.5, 87.1)*	76.6 (75.6, 85.6)*†	85.9 (85.0, 93.2)*	39.3 (39.2, 51.0)	81.9 (80.8, 85.7)*	78.1 (77.1, 88.3)*	92.0 (91.3, 96.3)*
Right Adrenal Gland	CT	32.1 (24.5, 42.0)	73.0 (61.9, 82.3)*†	64.4 (53.9, 70.4)*†	75.7 (71.5, 80.2)*	50.7 (42.6, 57.9)	85.8 (73.1, 93.2)*†	78.1 (70.8, 87.0)*†	93.8 (89.1, 96.3)*
Right Kidney	CT	89.8 (82.2, 93.8)	97.2 (94.2, 98.1)*†	93.6 (92.0, 95.0)*†	95.1 (94.1, 97.1)*	81.0 (69.7, 88.1)	97.0 (93.0, 98.9)*	93.2 (89.5, 96.1)*†	97.6 (94.3, 98.9)*
Right Lung	CT	95.1 (93.8, 96.3)	98.4 (98.1, 98.7)*†	96.7 (96.0, 97.2)*†	98.2 (97.9, 98.4)*	76.9 (72.8, 81.9)	97.0 (96.2, 97.7)*†	89.9 (87.5, 92.4)*†	95.8 (94.4, 96.9)*
Spinal Cord	CT	8.2 (8.4, 14.7)	73.3 (73.4, 81.5)*	75.8 (76.4, 78.2)*	75.5 (77.2, 80.3)*	30.0 (30.4, 32.8)	66.2 (66.4, 76.0)*	73.7 (73.8, 81.7)*	69.8 (77.7, 80.3)*
Spleen	CT	82.3 (51.2, 92.4)	97.4 (96.3, 98.6)*†	94.5 (92.9, 95.7)*†	96.4 (95.3, 97.4)*	65.4 (54.5, 78.3)	98.9 (96.0, 99.7)*	95.0 (89.6, 97.5)*†	98.5 (96.4, 99.6)*
Stomach	CT	76.1 (66.7, 83.4)	94.9 (93.2, 96.8)*	90.7 (87.6, 92.6)*†	95.0 (93.5, 96.4)*	60.9 (48.4, 72.7)	93.3 (86.7, 97.2)*	83.1 (72.8, 90.3)*†	94.2 (89.8, 97.5)*
Throat Cancer	CT	21.7 (26.7, 67.7)	78.7 (80.0, 88.0)*	72.3 (72.4, 87.0)*	84.0 (87.6, 94.3)	67.0 (70.3, 85.6)	87.9 (88.3, 96.9)*	88.7 (91.3, 91.8)*	92.0 (94.1, 99.8)

TABLE 7
Internal validation results of SAM, specialist U-Net, specialist DeepLabV3+, and MedSAM on MR image segmentation tasks in terms of DSC and NSD. Scores are displayed as Median values (First quartile, Third quartile). The Wilcoxon signed-rank test was performed for the validation results of each target. Scores marked with $*$ indicate statistically significant superiority over SAM, while those marked with † denote statistically significant superiority of MedSAM over the specialist U-Net model (p-value $<$ 0.05). Source data are provided as a Source Data file.

Target	Modality	DSC (%)				NSD (%)			
		SAM	U-Net	DeepLabV3+	MedSAM	SAM	U-Net	DeepLabV3+	MedSAM
Aorta	MR	78.4 (77.3, 87.3)	89.8 (89.5, 94.4)*†	90.1 (89.6, 93.2)*†	93.8 (93.6, 94.2)*	64.5 (63.9, 80.2)	90.6 (88.2, 98.2)*†	92.9 (91.8, 96.6)*†	96.8 (96.7, 98.7)*
Brainstem	MR	70.9 (69.8, 80.0)	86.3 (86.2, 87.5)*†	88.3 (88.2, 90.8)*†	95.2 (95.2, 95.9)*	83.6 (82.8, 91.2)	97.5 (97.5, 99.3)*†	97.9 (97.3, 99.7)*†	100.0 (100.0, 100.0)*
Cerebellum	MR	79.8 (79.5, 87.6)	90.3 (90.1, 93.8)*†	91.1 (90.9, 92.9)*†	95.1 (95.0, 95.6)*	87.3 (85.8, 92.1)	99.6 (99.4, 100.0)*	99.4 (99.4, 100.0)*†	100.0 (100.0, 100.0)*
Deep Grey Matter	MR	59.9 (58.5, 63.1)	88.9 (88.8, 93.3)*†	81.8 (80.2, 90.4)*†	93.6 (93.5, 94.7)*	70.1 (69.9, 79.5)	99.0 (98.6, 99.7)*	90.7 (88.7, 96.8)*†	98.3 (98.1, 99.9)*
Esophagus	MR	51.9 (51.3, 71.2)	72.1 (71.8, 82.0)*†	74.5 (73.6, 89.3)*	79.0 (78.6, 94.0)*	59.3 (58.3, 67.6)	84.7 (83.9, 89.4)*†	85.7 (84.9, 95.3)*	88.5 (87.8, 99.8)*
Gallbladder	MR	85.8 (82.5, 88.0)	90.1 (89.9, 93.0)*†	87.8 (87.3, 91.2)*†	93.8 (93.4, 94.8)*	85.5 (82.2, 90.8)	94.0 (92.4, 98.7)*†	91.8 (90.9, 97.6)*†	98.8 (98.4, 99.4)*
Glioma Enhancing Tumor T2	MR	74.7 (69.3, 79.6)	84.3 (80.4, 87.7)*†	82.3 (77.3, 86.3)*†	87.0 (83.6, 89.9)*	56.1 (49.4, 66.8)	79.1 (73.0, 83.6)*†	75.0 (67.2, 83.5)*†	86.8 (84.0, 92.5)*
Glioma FLAIR	MR	81.1 (76.2, 85.3)	93.2 (90.8, 94.8)*	92.5 (90.6, 94.4)*	93.0 (90.7, 94.3)*	64.4 (56.7, 73.3)	94.3 (90.5, 96.8)*	94.7 (90.0, 97.4)*	95.4 (91.7, 97.8)*
Glioma T1	MR	73.2 (67.5, 78.2)	87.6 (84.4, 90.7)*†	81.8 (77.6, 85.6)*†	89.1 (85.9, 91.1)*	52.0 (46.5, 56.2)	82.1 (75.8, 87.3)*†	70.2 (63.1, 76.1)*†	86.3 (82.3, 90.8)*
Inferior Vena Cava	MR	70.0 (68.4, 76.2)	81.5 (80.6, 84.5)*†	83.7 (82.2, 86.3)*†	89.4 (88.5, 92.0)*	66.2 (61.8, 73.6)	84.2 (82.5, 88.9)*†	88.1 (87.1, 91.2)*†	95.8 (94.6, 97.3)*
Ischemic Stroke ADC	MR	76.0 (73.6, 81.5)	85.5 (84.4, 87.9)*†	90.1 (87.5, 92.9)*	91.3 (90.2, 93.0)*	72.9 (70.9, 77.6)	85.3 (84.1, 89.8)*†	94.6 (90.5, 97.6)*†	98.3 (97.5, 99.2)*
Ischemic Stroke DWI	MR	85.3 (82.1, 88.5)	90.7 (88.8, 92.4)*†	93.3 (92.8, 95.7)*	92.2 (91.5, 93.9)*	87.1 (78.9, 90.1)	91.6 (88.7, 95.8)*†	98.0 (96.7, 99.4)*†	99.3 (98.8, 99.5)*
Left Atrium	MR	80.3 (80.2, 81.7)	90.6 (90.3, 91.5)*†	92.0 (91.8, 93.7)*†	94.8 (94.6, 95.6)*	55.5 (54.8, 58.1)	83.1 (82.7, 86.1)*†	89.1 (88.4, 95.8)*†	97.2 (97.1, 98.4)*
Left Kidney	MR	90.6 (90.4, 91.1)	96.7 (96.6, 97.3)*	94.6 (94.4, 94.9)*†	96.2 (96.1, 96.6)*	83.3 (82.9, 88.8)	99.1 (98.6, 99.7)*	96.3 (96.1, 98.3)*†	99.3 (99.3, 99.7)*
Left Ventricle	MR	76.1 (73.3, 80.0)	94.0 (93.9, 95.3)*†	93.5 (93.2, 96.7)*†	97.6 (96.6, 98.3)*	69.1 (68.6, 74.3)	98.1 (97.9, 98.8)*†	97.6 (96.1, 99.0)*†	99.5 (99.3, 99.9)*
Liver	MR	89.4 (88.8, 92.4)	97.4 (97.2, 98.1)*	94.7 (94.0, 95.6)*†	97.1 (96.9, 97.7)*	68.9 (60.0, 79.7)	97.0 (95.7, 98.7)*	88.0 (85.7, 92.9)*†	96.3 (95.5, 98.1)*
Meningioma T1-CE	MR	93.7 (93.6, 95.0)	95.2 (95.0, 97.3)	94.6 (94.6, 96.8)	95.4 (95.1, 97.6)*	93.9 (93.1, 99.7)	98.2 (97.7, 99.7)	98.8 (98.7, 99.8)*	99.6 (99.6, 99.9)*
Meningiomai T2-FLAIR	MR	80.3 (78.8, 87.8)	90.7 (90.6, 93.6)*	92.8 (91.8, 94.9)*	93.0 (92.9, 95.1)*	55.5 (54.8, 86.5)	88.5 (88.4, 94.2)*	94.0 (93.7, 99.6)*	94.9 (94.5, 97.3)*
Prostate	MR	90.7 (89.3, 91.5)	95.5 (95.2, 95.7)*†	94.5 (94.2, 95.2)*†	97.2 (97.0, 97.4)*	94.0 (92.2, 97.0)	99.6 (99.3, 99.8)*†	98.8 (98.4, 99.1)*†	99.8 (99.7, 99.9)*
Prostate ADC	MR	84.6 (80.3, 88.0)	90.4 (89.9, 92.1)*†	94.5 (92.0, 95.6)*†	95.7 (95.2, 96.7)*	79.1 (74.7, 82.1)	94.2 (91.6, 97.6)*†	97.9 (97.1, 98.9)*†	98.5 (98.2, 99.1)*
Right Kidney	MR	90.2 (90.0, 93.6)	96.3 (95.8, 97.1)*	93.2 (93.0, 94.8)*†	95.6 (95.5, 96.4)*	85.8 (84.6, 93.0)	98.3 (97.5, 99.5)*†	95.8 (95.4, 98.1)*†	99.0 (98.8, 99.5)*
Right Ventricle	MR	91.5 (89.6, 93.9)	95.0 (94.6, 95.7)*†	93.2 (92.8, 95.7)*†	96.7 (96.5, 97.8)*	92.0 (89.5, 97.8)	98.3 (98.2, 99.0)*†	97.5 (96.6, 99.2)*†	99.6 (99.4, 99.8)*
Spine	MR	73.6 (72.5, 78.3)	92.9 (92.1, 93.8)*†	83.5 (82.8, 85.8)*†	91.4 (90.7, 92.3)*	84.7 (83.6, 86.0)	99.2 (99.1, 99.3)*†	94.7 (94.1, 96.1)*†	98.7 (98.6, 98.9)*
Spleen	MR	89.7 (88.8, 93.1)	95.9 (95.8, 96.5)*	94.2 (94.0, 95.0)*†	95.9 (95.7, 96.5)*	80.4 (78.8, 94.5)	95.9 (94.7, 99.0)*†	94.5 (92.4, 98.4)*†	98.3 (97.9, 99.0)*
Stomach	MR	76.1 (75.9, 85.0)	92.2 (92.0, 95.0)*†	88.2 (87.5, 89.6)*†	94.1 (93.4, 95.1)*	59.3 (57.8, 79.0)	94.3 (92.9, 97.3)*	83.1 (81.0, 90.5)*†	95.8 (95.1, 98.3)*
Stroke T1	MR	69.8 (59.7, 80.7)	80.7 (78.7, 84.6)*†	89.5 (87.7, 90.9)*†	82.8 (81.4, 85.3)*	80.7 (71.5, 90.9)	95.2 (91.4, 97.5)*†	99.8 (99.5, 100.0)*†	96.7 (94.1, 98.7)*
Ventricles	MR	64.1 (63.7, 74.0)	89.6 (89.5, 93.3)*	72.9 (71.8, 85.4)†	89.3 (89.2, 92.4)*	78.3 (78.2, 79.9)	99.2 (99.1, 99.8)*†	79.7 (79.1, 84.1)†	89.9 (89.5, 95.0)*
Vestibular Schwannoma	MR	89.9 (86.7, 93.4)	91.6 (90.4, 93.6)†	91.2 (90.5, 93.9)†	96.5 (96.0, 97.6)*	98.0 (97.6, 99.2)	99.4 (99.2, 100.0)*†	99.9 (99.8, 100.0)*†	100.0 (100.0, 100.0)*

TABLE 8

Internal validation results of SAM, specialist U-Net, specialist DeepLabV3+, and MedSAM on CXR, Mammography, OCT, Ultrasound, Dermoscopy, Endoscopy, Fundus, and Pathology image segmentation tasks in terms of DSC and NSD. Scores are displayed as Median values (First quartile, Third quartile). The Wilcoxon signed-rank test was performed for the validation results of each target. Scores marked with $*$ indicate statistically significant superiority over SAM, while those marked with † denote statistically significant superiority of MedSAM over the specialist models (p-value < 0.05). Source data are provided as a Source Data file.

Target	Modality	DSC (%)				NSD (%)			
		SAM	U-Net	DeepLabV3+	MedSAM	SAM	U-Net	DeepLabV3+	MedSAM
COVID-19	CXR	76.6 (68.5, 84.1)	89.7 (85.2, 94.1)*†	92.2 (88.2, 94.7)*†	92.3 (88.7, 95.3)*	85.0 (78.9, 90.7)	96.7 (94.3, 98.5)*†	98.5 (96.0, 99.4)*	98.1 (96.4, 99.3)*
Heart	CXR	90.6 (89.3, 95.0)	96.3 (95.4, 97.2)*†	95.4 (94.6, 96.2)*†	97.2 (96.7, 98.0)*	95.2 (94.0, 98.3)	98.9 (98.1, 99.6)*†	98.4 (97.8, 99.0)*†	99.5 (99.2, 99.8)*
Left Lung	CXR	94.9 (92.1, 96.4)	98.2 (97.4, 98.8)*†	97.7 (97.1, 98.1)*†	98.6 (98.1, 98.9)*	98.3 (96.3, 99.3)	99.9 (98.6, 100.0)*†	99.9 (98.7, 100.0)*†	100.0 (99.9, 100.0)*
Pneumonia	CXR	88.9 (84.9, 92.2)	96.9 (95.8, 97.8)*†	96.9 (95.9, 97.5)*†	97.4 (96.4, 98.1)*	93.4 (90.1, 96.8)	99.6 (99.1, 99.9)*†	99.8 (99.2, 99.9)*	99.8 (99.4, 100.0)*
Pneumothorax	CXR	58.9 (46.6, 71.7)	89.8 (86.1, 92.6)*†	94.9 (93.5, 96.3)*	94.4 (93.7, 95.2)*	64.3 (52.9, 75.4)	94.0 (90.9, 96.2)*†	98.4 (97.7, 99.0)*	98.2 (97.6, 98.7)*
Right Lung	CXR	92.9 (87.9, 95.1)	97.9 (97.0, 98.6)*†	97.7 (97.0, 98.2)*†	98.4 (97.8, 98.8)*	96.9 (92.5, 98.8)	99.9 (98.6, 100.0)*†	99.9 (98.6, 100.0)*†	100.0 (99.9, 100.0)*
Breast Cancer	Mammography	63.7 (46.4, 77.9)	82.8 (76.1, 88.2)*	81.1 (74.7, 86.7)*†	83.4 (79.8, 88.5)*	78.4 (60.6, 88.4)	85.8 (79.8, 90.5)*†	83.3 (79.1, 88.7)*†	94.7 (91.7, 97.9)*
Diabetic Macular Edema	OCT	88.4 (82.3, 91.5)	89.7 (87.5, 92.8)*†	86.9 (83.2, 89.8)†	94.8 (93.2, 96.3)*	98.5 (95.5, 100.0)	96.6 (95.1, 98.2)*†	97.3 (94.7, 98.7)*†	100.0 (100.0, 100.0)*
Benign Breast Cancer	US	89.1 (82.5, 92.8)	89.9 (86.3, 93.1)†	92.2 (90.1, 94.9)*†	94.1 (92.3, 95.4)*	95.7 (88.5, 99.0)	93.5 (92.0, 95.9)†	95.8 (94.1, 97.6)†	98.4 (97.7, 99.6)*
Benign Thyroid Nodule	US	88.2 (83.2, 91.8)	93.3 (89.1, 95.7)*†	93.0 (90.8, 95.5)*†	95.1 (93.2, 96.9)*	93.3 (89.3, 96.1)	95.4 (92.7, 97.1)*†	95.8 (93.4, 97.0)*†	98.7 (97.7, 99.5)*
Fetal Head	US	86.2 (81.7, 89.7)	94.1 (93.4, 94.9)*†	98.5 (97.6, 99.0)*	98.3 (97.7, 98.8)*	91.7 (87.4, 94.5)	97.7 (97.0, 98.3)*†	100.0 (99.8, 100.0)*	100.0 (99.8, 100.0)*
Kidney	US	89.4 (84.8, 93.4)	97.1 (95.8, 97.9)*†	97.3 (96.2, 97.9)*†	97.8 (96.9, 98.4)*	93.9 (88.8, 97.2)	99.4 (98.7, 99.7)*†	99.8 (99.3, 99.9)*†	99.8 (99.6, 99.9)*
Left Atrium	US	84.1 (78.1, 87.4)	94.8 (94.1, 95.5)*†	98.6 (98.3, 98.8)*†	98.3 (97.9, 98.6)*	91.6 (88.0, 94.2)	97.5 (97.0, 98.0)*†	99.9 (99.8, 100.0)*†	100.0 (100.0, 100.0)*
Left Ventricle	US	19.5 (1.9, 83.0)	93.4 (92.4, 94.5)*†	98.4 (98.0, 98.7)*†	98.3 (97.8, 98.6)*	39.6 (4.9, 92.5)	96.5 (95.7, 97.2)*†	99.9 (99.8, 100.0)*†	100.0 (100.0, 100.0)*
Malignant Breast Cancer	US	86.1 (83.6, 89.9)	93.2 (86.5, 94.4)*†	90.9 (90.0, 95.6)*	94.0 (93.0, 96.9)*	91.2 (88.9, 94.1)	94.9 (89.6, 96.1)†	93.8 (92.8, 97.6)*†	98.5 (96.6, 98.9)*
Malignant Thyroid Nodule	US	88.0 (82.9, 91.8)	88.9 (83.7, 91.8)*†	90.1 (87.4, 92.7)*†	93.2 (91.4, 95.1)*	95.3 (90.7, 97.8)	92.6 (89.4, 95.0)*†	93.7 (91.3, 95.8)†	98.9 (97.6, 99.6)*
Nerve Cancer	US	57.7 (40.8, 70.5)	86.0 (84.0, 88.2)*†	89.0 (86.0, 91.3)*†	90.9 (88.5, 92.3)*	75.5 (61.8, 84.5)	90.8 (89.0, 92.6)*†	93.6 (90.6, 95.5)*†	97.6 (96.3, 98.6)*
Pubic Symphysis	US	69.6 (69.0, 79.0)	89.1 (88.1, 90.8)*†	94.5 (92.7, 97.4)*	93.5 (93.0, 95.5)*	82.7 (80.6, 90.7)	96.3 (95.8, 97.5)*†	99.9 (99.4, 100.0)*	99.5 (99.2, 100.0)*
Skin Cancer	Dermoscopy	90.1 (84.9, 93.9)	94.3 (92.1, 96.1)*†	94.8 (92.6, 96.3)*†	96.4 (95.0, 97.7)*	96.1 (90.9, 98.7)	96.1 (94.2, 97.6)*†	96.3 (94.4, 97.7)*†	99.7 (98.9, 99.9)*
Cholecystectomy	Endoscopy	49.8 (33.8, 62.8)	49.2 (38.2, 57.7)†	53.2 (44.3, 59.0)*†	87.7 (70.4, 93.5)*	55.4 (38.2, 66.9)	54.5 (43.4, 62.0)†	57.5 (49.1, 62.5)*†	92.2 (73.9, 96.5)*
Gastrectomy	Endoscopy	66.8 (55.7, 76.3)	80.6 (71.6, 86.0)*†	71.9 (62.8, 79.4)*†	88.1 (79.2, 92.8)*	71.4 (60.3, 80.4)	84.3 (75.6, 89.7)*†	75.8 (66.6, 83.2)*†	92.2 (83.1, 96.4)*
Polyp	Endoscopy	91.3 (81.2, 95.1)	94.0 (90.3, 96.0)*†	92.5 (88.5, 95.3)*†	97.9 (96.7, 98.5)*	95.7 (85.0, 98.8)	95.7 (92.3, 97.3)*†	94.2 (90.1, 96.8)†	99.3 (98.7, 100.0)*
Surgical Instrument	Endoscopy	85.0 (81.7, 91.3)	89.3 (83.6, 94.3)*†	80.2 (70.4, 89.7)*†	95.1 (93.9, 96.8)*	88.3 (85.2, 93.3)	92.1 (86.0, 96.2)†	83.8 (73.6, 91.8)*†	97.5 (96.7, 98.3)*
Glaucoma Cup	Fundus	63.6 (58.3, 72.0)	91.1 (90.5, 91.9)*†	90.3 (88.5, 94.4)*†	96.3 (95.5, 97.2)*	66.3 (61.5, 74.4)	93.6 (93.0, 94.3)*†	92.6 (92.0, 97.1)*†	98.6 (98.5, 99.3)*
Glaucoma Disc	Fundus	78.7 (72.3, 91.4)	94.9 (94.8, 95.8)*†	96.6 (95.8, 97.3)*†	97.8 (97.7, 98.2)*	81.3 (75.3, 93.1)	96.6 (96.5, 97.2)*†	98.1 (97.6, 98.8)*†	99.2 (99.1, 99.4)*
Non-Glaucoma Cup	Fundus	52.6 (42.4, 66.3)	88.0 (87.1, 89.5)*†	90.9 (88.1, 92.9)*†	95.6 (94.9, 96.3)*	56.9 (46.4, 69.9)	91.7 (90.6, 92.7)*†	94.4 (91.4, 96.2)*†	98.7 (98.3, 99.1)*
Non-Glaucoma Disc	Fundus	62.5 (54.1, 73.1)	94.5 (93.8, 95.5)*†	95.9 (94.7, 97.0)*†	97.5 (97.2, 98.0)*	65.5 (57.4, 76.4)	96.2 (95.7, 97.1)*†	97.6 (96.4, 98.4)*†	99.2 (98.9, 99.4)*
Glomeruli	Pathology	91.1 (87.3, 93.8)	95.4 (94.2, 96.3)*†	94.9 (93.0, 96.2)*†	97.5 (97.1, 97.9)*	96.8 (94.0, 98.6)	97.0 (95.7, 97.7)*†	96.6 (94.4, 97.5)†	99.9 (99.7, 100.0)*
Intestine FTU	Pathology	89.1 (87.4, 92.2)	92.6 (92.0, 93.8)*†	92.3 (90.5, 94.4)*†	95.9 (95.5, 96.7)*	96.9 (95.3, 98.5)	95.9 (95.2, 96.4)†	95.3 (93.6, 96.8)†	99.8 (99.6, 100.0)*

TABLE 9

External validation results of SAM, specialist U-Net, specialist DeepLabV3+, and MedSAM on CT image segmentation tasks in terms of DSC and NSD. Scores are displayed as Median values (First quartile, Third quartile). The Wilcoxon signed-rank test was performed for the validation results of each target. Scores marked with $*$ indicate statistically significant superiority over SAM, while those marked with † denote statistically significant superiority of MedSAM over the specialist models (p-value < 0.05). Source data are provided as a Source Data file.

Target	Modality	DSC (%)				NSD (%)			
		SAM	U-Net	DeepLabV3+	MedSAM	SAM	U-Net	DeepLabV3+	MedSAM
Adrenocortical carcinoma	CT	86.1 (80.2, 89.5)	91.0 (89.8, 93.7)*†	90.8 (89.3, 92.6)*†	92.7 (91.2, 94.7)*	75.3 (62.6, 85.2)	92.1 (80.6, 96.1)*†	87.6 (77.4, 93.9)*†	93.8 (88.9, 97.5)*
Aorta	CT	77.8 (70.8, 81.9)	92.8 (90.6, 95.0)*†	89.5 (87.3, 90.9)*†	94.3 (93.6, 94.8)*	58.2 (52.7, 63.8)	90.8 (87.2, 94.9)*†	92.3 (87.6, 95.2)*†	98.4 (97.8, 98.9)*
Brainstem	CT	38.8 (35.4, 42.8)	78.7 (76.6, 80.4)*	67.9 (66.2, 72.5)*†	77.5 (74.5, 81.5)*	27.4 (25.7, 33.3)	73.4 (66.1, 81.5)*	54.6 (48.7, 63.4)*†	71.8 (64.3, 76.7)*
Esophagus	CT	54.5 (47.9, 60.2)	81.4 (76.2, 85.3)*	76.4 (71.9, 79.6)*†	80.4 (76.9, 85.0)*	54.8 (49.4, 62.1)	91.5 (87.0, 94.2)*	88.6 (84.5, 92.6)*†	91.9 (88.4, 95.1)*
Esophagus	CT	32.8 (27.8, 38.8)	64.1 (60.6, 67.2)*†	55.4 (47.7, 60.1)*†	49.8 (43.8, 57.1)*	38.6 (35.9, 42.5)	68.3 (63.9, 74.1)*†	58.4 (51.1, 65.5)*	54.1 (49.2, 61.9)*
Gallbladder	CT	63.7 (53.7, 75.5)	85.5 (79.5, 89.1)*	78.1 (69.6, 84.2)*†	86.6 (78.7, 90.2)*	67.2 (59.7, 75.4)	93.3 (90.2, 96.6)*	86.1 (79.5, 91.3)*†	95.0 (87.8, 97.8)*
Heart	CT	89.6 (88.4, 90.5)	89.3 (88.5, 90.6)*†	88.4 (87.2, 89.6)*†	96.4 (96.3, 96.6)*	73.3 (71.2, 77.0)	80.5 (79.2, 82.9)*†	69.1 (65.7, 73.0)*†	96.4 (96.0, 97.5)*
Hepatocellular carcinoma	CT	64.2 (49.4, 80.5)	87.8 (84.6, 91.6)*†	86.5 (83.3, 90.1)*†	95.0 (93.4, 96.3)*	55.3 (50.6, 60.9)	83.0 (78.4, 88.4)*†	79.9 (75.1, 85.8)*†	96.9 (95.5, 98.2)*
Inferior Vena Cava	CT	56.1 (49.8, 61.8)	80.4 (75.8, 83.6)*†	75.6 (69.5, 78.4)*†	84.8 (81.2, 87.5)*	44.2 (40.4, 47.2)	74.2 (70.3, 78.1)*†	68.5 (63.3, 73.1)*†	85.7 (81.8, 89.4)*
Left Adrenal Gland	CT	43.5 (38.2, 49.5)	74.4 (70.7, 78.2)*†	62.0 (56.2, 66.3)*†	70.0 (62.5, 74.4)*	51.1 (47.6, 56.2)	88.5 (84.9, 93.0)*†	78.4 (70.1, 84.0)*†	85.2 (75.8, 90.3)*
Left Kidney	CT	87.9 (84.4, 90.9)	93.7 (92.9, 94.6)*†	91.7 (90.8, 92.6)*†	95.6 (95.0, 95.9)*	75.4 (68.6, 81.3)	92.9 (91.3, 95.0)*†	90.0 (87.8, 92.4)*†	98.6 (97.8, 99.0)*
Left Lung	CT	89.1 (87.7, 92.6)	94.5 (93.0, 95.5)*†	92.7 (90.6, 94.0)*†	96.2 (95.8, 96.9)*	74.8 (69.2, 80.0)	89.9 (87.2, 93.1)*†	80.4 (76.7, 88.4)*†	96.1 (94.9, 96.9)*
Liver	CT	87.5 (83.2, 90.2)	95.6 (94.9, 96.3)*†	93.4 (92.3, 94.3)*†	96.7 (96.2, 97.1)*	64.6 (57.7, 71.1)	93.8 (91.6, 95.6)*†	84.9 (81.0, 88.8)*†	96.3 (94.7, 97.4)*
Lung tumor	CT	41.5 (35.8, 52.0)	66.9 (62.3, 74.8)*	63.4 (59.1, 76.4)*†	74.8 (61.6, 88.7)*	46.8 (44.6, 54.5)	70.4 (65.3, 79.5)*†	67.4 (60.2, 74.7)*†	77.1 (68.1, 90.1)*
Lymph node	CT	54.7 (44.2, 62.9)	76.6 (71.3, 81.3)*†	78.6 (73.4, 83.7)*†	81.2 (75.7, 85.2)*	58.5 (52.1, 65.3)	87.5 (82.5, 92.0)*†	88.5 (83.3, 93.1)*†	90.5 (84.3, 95.2)*
Nasopharynx cancer	CT	35.5 (28.7, 40.6)	72.3 (70.4, 75.1)*†	65.1 (61.5, 70.4)*†	87.8 (85.0, 91.4)*	51.0 (44.3, 57.7)	71.4 (67.7, 79.4)*†	65.0 (59.7, 71.8)*†	92.0 (87.7, 95.3)*
Pancreas	CT	62.0 (55.4, 68.6)	83.0 (80.4, 85.6)*†	68.8 (62.1, 73.5)*†	87.0 (83.8, 89.2)*	67.1 (62.0, 71.4)	90.5 (87.1, 92.9)*†	72.7 (65.9, 78.2)*†	93.8 (89.5, 96.5)*
Parotid L	CT	22.4 (16.9, 33.0)	75.9 (73.1, 79.4)*†	66.1 (63.0, 71.0)*†	83.1 (80.2, 87.0)*	40.7 (35.9, 46.4)	73.5 (68.9, 76.7)*†	63.5 (57.9, 69.9)*†	83.7 (76.2, 89.6)*
Parotid R	CT	24.2 (20.7, 31.1)	74.5 (71.0, 79.4)*†	66.8 (61.3, 69.4)*†	81.2 (77.7, 86.9)*	41.7 (38.8, 50.1)	72.2 (68.5, 75.9)*†	61.2 (56.4, 69.9)*†	82.0 (75.1, 88.7)*
Right Adrenal Gland	CT	32.4 (25.5, 39.7)	67.1 (60.8, 72.6)*†	58.6 (53.1, 65.3)*†	70.8 (64.8, 75.5)*	49.2 (44.5, 55.2)	80.9 (75.1, 87.1)*†	74.1 (68.1, 82.9)*†	88.7 (82.1, 93.7)*
Right Kidney	CT	88.4 (85.9, 90.6)	94.0 (93.1, 94.7)*†	90.2 (89.1, 91.4)*†	95.3 (95.0, 95.7)*	75.3 (70.2, 81.9)	93.7 (91.5, 95.1)*†	84.0 (80.7, 87.9)*†	98.5 (98.2, 98.9)*
Right Lung	CT	90.0 (88.2, 93.4)	95.1 (94.4, 96.0)*†	92.3 (91.0, 93.7)*†	96.8 (96.6, 97.3)*	70.1 (60.9, 77.3)	90.3 (87.3, 92.6)*†	76.5 (71.9, 83.1)*†	96.3 (95.5, 97.1)*
Spleen	CT	84.1 (78.8, 89.2)	95.2 (94.1, 95.8)*†	91.5 (89.8, 92.8)*†	95.7 (95.3, 96.1)*	75.2 (68.3, 80.7)	98.0 (96.5, 99.0)*†	90.7 (86.9, 93.7)*†	98.8 (98.3, 99.2)*
Stomach	CT	81.9 (76.3, 85.7)	93.3 (91.8, 94.6)*†	86.9 (84.0, 89.4)*†	95.3 (94.5, 96.0)*	68.2 (62.0, 73.8)	93.3 (89.6, 96.1)*†	76.0 (69.7, 82.5)*†	97.7 (95.6, 98.5)*

TABLE 10
External validation results of SAM, specialist U-Net, specialist DeepLabV3+, and MedSAM on MR image segmentation tasks in terms of DSC and NSD. Scores are displayed as Median values (First quartile, Third quartile). The Wilcoxon signed-rank test was performed for the validation results of each target. Scores marked with ∗ indicate statistically significant superiority over SAM, while those marked with † denote statistically significant superiority of MedSAM over the specialist models (p-value < 0.05). Source data are provided as a Source Data file.

Target	Modality	DSC (%)				NSD (%)			
		SAM	U-Net	DeepLabV3+	MedSAM	SAM	U-Net	DeepLabV3+	MedSAM
Brain lesion	MR	84.3 (79.2, 89.1)	84.4 (82.2, 88.5)†	84.1 (78.3, 89.3)†	96.3 (95.3, 97.4)*	85.2 (72.6, 96.9)	92.4 (89.3, 96.1)*†	90.7 (83.1, 98.3)*†	100.0 (100.0, 100.0)*
Brainstem	MR	79.6 (76.8, 82.4)	89.0 (86.7, 90.3)*†	85.5 (84.4, 89.4)*†	95.3 (95.0, 96.1)*	70.3 (66.0, 74.6)	90.5 (85.3, 94.0)*†	82.1 (78.7, 89.6)*†	99.1 (97.2, 99.9)*
Brainstem	MR	71.8 (68.9, 76.3)	89.7 (88.8, 90.8)*†	88.6 (87.6, 89.5)*†	95.1 (94.9, 95.5)*	73.7 (71.5, 79.7)	97.3 (96.6, 98.1)*†	94.9 (93.6, 95.7)*†	99.7 (99.5, 99.9)*
Cervical cancer	MR	74.5 (78.5, 82.6)	83.0 (84.9, 90.0)†	78.9 (83.1, 86.1)†	93.5 (93.6, 93.9)*	84.4 (85.4, 88.1)	94.0 (94.0, 95.4)†	89.1 (89.7, 95.8)†	99.6 (99.6, 100.0)*
Eye L	MR	74.2 (69.8, 78.4)	89.5 (87.9, 91.0)*†	77.9 (75.4, 82.3)*†	96.0 (95.6, 96.9)*	58.1 (52.2, 75.0)	96.2 (92.9, 98.9)*†	75.2 (69.2, 83.8)*†	100.0 (100.0, 100.0)*
Eye Posterior L	MR	67.6 (65.4, 70.6)	86.0 (85.5, 87.3)*†	76.3 (72.4, 79.7)*†	92.6 (91.7, 93.6)*	69.5 (65.5, 75.7)	96.8 (95.6, 98.4)*†	89.8 (87.2, 92.3)*†	99.7 (99.2, 99.9)*
Eye Posterior R	MR	70.1 (67.3, 72.8)	86.4 (84.9, 87.5)*†	78.2 (73.4, 82.3)*†	92.5 (91.7, 93.5)*	72.1 (67.5, 80.3)	97.0 (95.0, 98.4)*†	92.8 (90.3, 95.1)*†	99.5 (99.1, 99.8)*
Eye R	MR	73.7 (70.2, 77.9)	89.3 (87.8, 91.0)*†	78.1 (73.6, 82.8)*†	96.2 (95.5, 96.7)*	60.1 (52.8, 73.6)	96.5 (92.9, 98.5)*†	73.9 (67.5, 83.9)*†	100.0 (100.0, 100.0)*
Glottis	MR	32.4 (28.7, 41.3)	56.2 (52.8, 62.5)*†	44.4 (40.4, 52.8)*†	81.6 (77.0, 86.8)*	60.5 (56.1, 69.0)	79.1 (75.9, 84.1)*†	71.1 (65.9, 76.9)*†	98.2 (96.0, 99.4)*
Left Kidney	T1-Inphase	88.5 (86.0, 93.3)	88.0 (85.2, 91.1)†	87.3 (86.5, 91.1)†	96.1 (95.8, 97.3)*	94.5 (92.3, 98.2)	96.7 (94.1, 98.7)†	96.5 (95.1, 99.2)†	100.0 (100.0, 100.0)*
Left Kidney	T1-Outphase	88.2 (85.8, 92.1)	85.9 (84.4, 89.3)†	89.4 (87.8, 90.8)†	96.3 (95.8, 97.2)*	94.7 (92.1, 98.4)	92.5 (90.3, 97.0)†	96.8 (93.7, 99.0)†	100.0 (100.0, 100.0)*
Left Kidney	T2	92.3 (90.8, 94.0)	94.2 (93.0, 95.4)*†	92.9 (92.5, 93.7)†	97.1 (96.1, 97.6)*	96.7 (94.8, 99.1)	98.4 (97.4, 99.6)†	98.9 (97.5, 99.9)*†	99.9 (99.8, 100.0)*
Left ventricle cavity	MR	76.9 (70.5, 82.8)	89.3 (85.0, 92.5)*†	90.2 (87.8, 92.9)*†	96.6 (95.7, 97.2)*	68.8 (61.9, 78.0)	92.4 (87.3, 95.7)*†	92.8 (88.8, 95.7)*†	99.4 (99.1, 99.7)*
Lips	MR	58.5 (52.6, 65.6)	73.7 (67.9, 79.7)*†	65.6 (62.4, 73.0)*†	86.3 (84.0, 88.6)*	76.5 (70.9, 83.1)	91.4 (84.9, 94.5)*†	84.2 (78.2, 90.3)*†	98.4 (97.8, 99.2)*
Liver	T1-Inphase	88.6 (87.9, 90.3)	92.6 (92.3, 94.4)*†	89.3 (87.8, 90.9)†	96.9 (96.4, 97.1)*	88.0 (80.6, 91.4)	95.7 (94.3, 97.7)*†	89.5 (87.3, 93.4)†	99.7 (99.2, 99.8)*
Liver	T1-Outphase	90.5 (90.0, 92.4)	94.2 (93.6, 95.5)*†	91.0 (89.2, 92.9)†	96.5 (96.2, 97.6)*	92.2 (88.6, 94.7)	97.5 (96.6, 98.7)*†	94.5 (88.7, 97.4)†	99.5 (99.0, 99.9)*
Liver	T2	89.8 (88.8, 90.7)	95.0 (94.8, 96.0)*†	90.3 (89.4, 91.3)†	97.1 (96.6, 97.5)*	89.9 (87.7, 93.4)	98.1 (97.5, 99.0)*†	92.0 (87.7, 94.2)†	99.6 (99.4, 99.9)*
Mandible Bone	MR	47.0 (44.6, 52.0)	24.7 (21.5, 29.2)*†	18.0 (14.4, 22.9)*†	73.9 (70.8, 78.8)*	48.3 (45.5, 52.2)	46.7 (43.5, 49.0)*†	39.2 (37.0, 42.2)*†	63.7 (62.0, 70.8)*
Parotid L	MR	69.1 (63.2, 73.8)	85.7 (84.3, 87.5)*†	76.0 (72.4, 80.1)*†	90.8 (90.4, 92.1)*	71.7 (65.3, 74.3)	93.1 (89.5, 95.9)*†	82.3 (78.0, 88.8)*†	98.5 (97.9, 99.0)*
Parotid R	MR	66.7 (62.0, 71.0)	84.3 (82.4, 86.6)*†	74.3 (69.7, 79.9)*†	90.8 (90.1, 91.5)*	72.2 (69.6, 77.7)	91.1 (88.2, 93.1)*†	82.2 (75.9, 87.7)*†	98.4 (97.7, 98.9)*
Prostate	MR	90.0 (86.2, 94.1)	95.0 (94.0, 96.4)*†	90.4 (87.2, 93.0)†	97.4 (97.2, 97.9)*	92.7 (88.0, 96.0)	97.5 (96.7, 98.4)*†	93.1 (89.9, 94.9)†	99.4 (99.2, 99.7)*
Right Kidney	T1-Inphase	90.1 (86.4, 92.7)	85.3 (83.1, 89.7)†	86.4 (84.9, 89.3)†	95.9 (94.7, 97.2)*	95.9 (94.2, 98.7)	96.8 (94.5, 98.2)†	95.4 (93.1, 97.6)†	100.0 (99.9, 100.0)*
Right Kidney	T1-Outphase	87.1 (84.8, 90.3)	88.1 (85.4, 90.3)†	88.4 (87.0, 90.7)†	96.3 (95.4, 97.2)*	92.4 (91.2, 98.8)	95.1 (93.4, 97.5)†	96.7 (95.7, 99.1)*†	100.0 (100.0, 100.0)*
Right Kidney	T2	93.4 (92.9, 94.0)	94.7 (92.8, 95.5)†	92.9 (92.7, 94.1)†	96.9 (96.2, 97.9)*	98.4 (97.8, 99.6)	98.9 (98.3, 99.5)†	98.9 (98.7, 99.6)†	100.0 (99.9, 100.0)*
Right ventricle cavity	MR	87.4 (81.9, 91.9)	90.3 (86.9, 92.6)*†	88.5 (84.9, 91.4)*†	95.4 (94.5, 96.2)*	91.4 (86.3, 95.1)	95.1 (92.6, 97.2)*†	92.9 (90.0, 95.4)*†	99.2 (98.9, 99.5)*
Spinal Cord	MR	39.5 (38.0, 41.1)	71.1 (70.1, 73.0)*	61.1 (55.6, 65.8)*†	70.4 (67.9, 75.5)*	33.9 (32.1, 41.3)	87.5 (85.5, 89.8)*†	68.6 (64.1, 74.4)*†	81.6 (78.7, 85.7)*
Spleen	T1-Inphase	85.1 (82.8, 87.7)	88.1 (86.7, 92.6)*†	81.4 (78.8, 85.0)*†	95.6 (94.9, 96.2)*	89.3 (83.7, 95.6)	97.4 (95.1, 98.2)*†	85.4 (81.4, 89.8)*†	100.0 (99.9, 100.0)*
Spleen	T1-Outphase	86.0 (84.7, 90.3)	90.9 (88.9, 92.3)*†	83.9 (82.1, 87.5)†	95.2 (94.9, 96.7)*	94.5 (92.3, 97.9)	98.0 (97.3, 99.7)*†	91.2 (90.5, 94.2)*†	99.9 (99.8, 100.0)*
Spleen	T2	93.8 (93.3, 95.3)	95.0 (94.6, 96.4)*†	92.2 (91.7, 94.4)†	97.0 (96.2, 97.9)*	99.7 (98.4, 100.0)	99.9 (99.7, 100.0)†	98.6 (96.1, 100.0)†	100.0 (100.0, 100.0)*
Supraglottic Larynx	MR	46.1 (42.4, 54.6)	70.1 (66.3, 73.7)*†	61.3 (59.2, 67.0)*†	86.3 (84.4, 88.9)*	76.4 (71.6, 80.9)	88.4 (85.3, 92.0)*†	87.7 (78.4, 91.6)*†	97.9 (97.2, 99.3)*

TABLE 11
External validation results of SAM, specialist U-Net, specialist DeepLabV3+, and MedSAM on CXR, Mammography, OCT, Ultrasound, Dermoscopy, Endoscopy, Fundus, and Pathology image segmentation tasks in terms of DSC and NSD. Scores are displayed as Median values (First quartile, Third quartile). The Wilcoxon signed-rank test was performed for the validation results of each target. Scores marked with ∗ indicate statistically significant superiority over SAM, while those marked with † denote statistically significant superiority of MedSAM over the specialist models (p-value < 0.05). Source data are provided as a Source Data file.

Target	Modality	DSC (%)				NSD (%)			
		SAM	U-Net	DeepLabV3+	MedSAM	SAM	U-Net	DeepLabV3+	MedSAM
COVID-19 Lung	CXR	91.0 (87.4, 93.9)	98.7 (98.1, 99.0)*†	98.1 (97.6, 98.5)*†	97.9 (97.3, 98.3)*	95.6 (92.1, 97.9)	99.9 (99.8, 100.0)*	100.0 (99.8, 100.0)*	99.9 (99.8, 100.0)*
Lung Opacity	CXR	88.6 (84.6, 91.7)	98.2 (97.4, 98.7)*†	97.7 (97.0, 98.2)*†	97.5 (96.7, 98.1)*	93.8 (90.3, 96.5)	99.8 (99.6, 99.9)*	99.8 (99.5, 100.0)*	99.8 (99.5, 100.0)*
Normal Lung	CXR	93.6 (90.7, 95.3)	99.3 (99.0, 99.4)*†	98.6 (98.4, 98.8)*	98.7 (98.3, 98.9)*	97.5 (94.6, 98.9)	100.0 (100.0, 100.0)*†	100.0 (100.0, 100.0)*†	100.0 (100.0, 100.0)*
Pneumonia Lung	CXR	87.0 (80.6, 91.5)	98.3 (97.4, 98.7)*†	97.7 (96.9, 98.1)*	97.6 (96.5, 98.1)*	92.2 (85.7, 96.3)	99.8 (99.6, 99.9)*	99.9 (99.6, 100.0)*	99.9 (99.5, 100.0)*
Skin Cancer	Dermoscopy	94.4 (91.9, 96.1)	92.7 (89.6, 95.0)*†	93.6 (90.9, 95.0)†	96.5 (95.7, 97.0)*	99.4 (97.1, 99.8)	94.9 (92.1, 96.7)*†	96.0 (94.2, 96.9)*†	99.9 (99.7, 100.0)*
Polyp	Endoscopy	93.9 (90.2, 95.8)	95.8 (93.5, 97.1)*†	95.2 (92.9, 96.8)*†	97.7 (96.9, 98.2)*	96.6 (93.4, 98.1)	97.9 (95.8, 98.9)*†	97.5 (95.2, 98.7)*†	99.4 (99.0, 99.7)*
Optic Cup	Fundus	56.0 (47.2, 65.5)	90.5 (85.4, 93.2)*†	86.8 (77.7, 90.8)*†	95.0 (92.8, 96.0)*	58.1 (50.1, 67.9)	93.0 (88.7, 95.2)*†	89.2 (80.3, 93.0)*†	97.5 (95.8, 98.1)*
Optic Disc	Fundus	87.4 (76.1, 92.9)	97.7 (96.8, 98.1)*†	96.2 (95.0, 97.1)*†	97.7 (97.3, 98.0)*	89.1 (78.6, 94.0)	98.5 (97.8, 98.9)*†	97.1 (95.8, 97.9)*†	98.6 (98.3, 98.9)*
Colon Gland	Pathology	80.4 (68.9, 87.0)	87.3 (82.0, 90.1)*†	79.3 (73.9, 84.2)†	95.6 (94.6, 96.3)*	84.7 (73.8, 91.3)	90.4 (84.6, 93.0)*†	82.7 (78.0, 87.6)†	98.2 (97.4, 98.7)*
Fetal Head	US	92.4 (79.9, 95.2)	97.2 (96.5, 97.7)	96.4 (95.2, 97.1)	97.3 (96.6, 97.9)	94.5 (86.0, 96.8)	98.2 (97.6, 98.6)	97.4 (96.3, 98.1)	98.4 (97.7, 98.8)

TABLE 12

Performance change with varying training data size on the internal validation set (CT and MR images) in terms of DSC and NSD scores. Scores for each task are summarized as Median values (First quartile, Third quartile). Source data are provided as a Source Data file.

Task	Modality	DSC			NSD		
		10K	100K	1M	10K	100K	1M
Aorta	CT	88.3 (84.7, 91.6)	91.4 (88.8, 94.5)	93.6 (92.4, 94.9)	87.6 (81.1, 92.4)	94.3 (89.4, 97.9)	98.2 (96.3, 99.1)
Colon Cancer	CT	81.2 (80.6, 86.0)	78.9 (78.4, 85.8)	85.4 (83.2, 88.5)	87.9 (81.0, 92.5)	86.9 (84.8, 90.1)	92.1 (91.7, 97.8)
COVID-19 Infection	CT	72.4 (68.3, 77.9)	80.0 (73.7, 82.8)	87.9 (86.6, 91.9)	80.1 (70.0, 85.6)	85.3 (81.6, 89.3)	94.7 (90.5, 97.4)
Esophagus	CT	78.6 (73.4, 82.1)	82.7 (78.9, 86.3)	85.2 (82.6, 88.1)	84.5 (79.2, 90.0)	92.1 (87.4, 95.8)	95.3 (92.9, 97.5)
Gallbladder	CT	82.0 (73.7, 86.9)	89.2 (82.7, 92.3)	91.5 (87.7, 94.4)	78.3 (69.2, 87.6)	92.6 (87.4, 96.9)	96.6 (92.8, 98.8)
Glioblastoma	CT	84.9 (83.8, 88.7)	84.3 (82.9, 86.9)	95.7 (95.3, 96.2)	77.2 (72.7, 83.8)	76.7 (72.1, 80.9)	96.7 (96.1, 98.4)
Head-Neck Cancer	CT	73.4 (71.3, 76.0)	76.4 (72.2, 79.2)	83.1 (79.5, 86.9)	63.9 (59.7, 72.9)	73.5 (69.5, 78.9)	83.3 (76.8, 88.3)
Heart	CT	91.9 (92.5, 93.7)	92.7 (93.7, 95.1)	95.0 (95.0, 96.6)	78.5 (80.0, 85.8)	81.4 (86.5, 88.5)	89.5 (89.9, 93.8)
Inferior Vena Cava	CT	82.8 (79.2, 85.3)	86.8 (83.2, 90.2)	89.8 (87.6, 92.0)	73.5 (68.9, 81.1)	84.9 (79.5, 91.3)	92.6 (89.0, 95.8)
Intracranial Hemorrhage	CT	87.8 (87.5, 91.4)	88.9 (88.1, 93.6)	95.2 (95.1, 96.7)	93.9 (93.1, 98.6)	96.6 (96.0, 98.6)	99.6 (99.6, 99.8)
Kidney Cancer	CT	88.4 (85.8, 90.8)	90.1 (87.4, 93.4)	95.0 (92.2, 96.7)	83.7 (79.3, 90.8)	88.6 (84.9, 92.2)	97.7 (96.2, 98.9)
Left Adrenal Gland	CT	68.6 (61.7, 74.1)	74.1 (68.3, 79.9)	79.2 (75.1, 83.7)	83.1 (76.8, 87.1)	88.3 (83.6, 92.8)	93.5 (89.9, 96.8)
Left Kidney	CT	92.3 (89.8, 94.6)	94.1 (92.8, 96.6)	95.3 (94.2, 97.1)	88.6 (83.6, 93.3)	95.1 (92.6, 98.0)	97.8 (94.6, 99.0)
Left Lung	CT	97.8 (97.5, 98.1)	97.9 (97.0, 98.3)	98.3 (98.0, 98.5)	93.3 (91.7, 94.6)	92.6 (86.7, 95.7)	96.2 (95.5, 97.2)
Liver	CT	94.0 (93.0, 95.2)	96.2 (95.4, 97.3)	97.2 (96.7, 97.7)	79.0 (72.8, 88.1)	92.8 (82.8, 96.6)	95.5 (91.9, 97.8)
Liver Cancer	CT	85.6 (78.5, 88.8)	86.3 (81.5, 90.7)	89.4 (83.4, 92.7)	82.9 (75.1, 88.5)	88.4 (84.6, 94.8)	92.7 (87.5, 95.9)
Lung Cancer	CT	81.8 (78.8, 86.7)	80.5 (74.0, 87.2)	93.8 (88.7, 95.1)	77.9 (74.7, 86.1)	83.2 (76.0, 87.6)	96.6 (92.7, 97.8)
Lymph Nodes	CT	75.6 (74.0, 80.1)	78.9 (76.4, 82.1)	85.0 (84.1, 86.6)	78.1 (75.4, 87.1)	85.7 (81.6, 88.9)	91.3 (90.4, 94.8)
Melanoma	CT	76.1 (71.5, 79.5)	75.4 (69.7, 79.3)	77.5 (73.1, 81.7)	63.8 (60.1, 69.8)	64.9 (61.6, 71.8)	69.1 (65.3, 75.2)
Pancreas	CT	73.1 (65.7, 80.3)	81.6 (76.5, 86.6)	85.5 (81.4, 89.1)	70.5 (59.9, 80.0)	82.0 (73.1, 90.3)	87.4 (80.8, 93.9)
Pancreas Cancer	CT	75.3 (68.5, 80.9)	73.5 (67.7, 81.1)	77.8 (74.5, 83.9)	78.3 (76.5, 85.6)	74.6 (70.1, 84.3)	84.5 (76.3, 90.5)
Pleural Effusion	CT	62.1 (60.0, 73.6)	74.3 (73.0, 83.5)	85.9 (85.0, 93.2)	68.0 (61.1, 75.9)	77.4 (76.7, 86.4)	92.0 (91.3, 96.3)
Right Adrenal Gland	CT	63.5 (57.8, 69.5)	69.3 (62.7, 77.4)	75.7 (71.5, 80.2)	78.5 (70.7, 83.9)	88.3 (81.2, 92.4)	93.8 (89.1, 96.3)
Right Kidney	CT	91.5 (89.7, 94.5)	93.8 (91.9, 96.7)	95.1 (94.1, 97.1)	88.0 (81.5, 93.2)	93.7 (90.1, 97.3)	97.6 (94.3, 98.9)
Right Lung	CT	97.6 (97.3, 98.0)	97.0 (96.2, 97.6)	98.2 (97.9, 98.4)	93.6 (91.0, 94.8)	87.8 (81.4, 93.0)	95.8 (94.4, 96.9)
Spinal Cord	CT	71.9 (72.2, 79.1)	67.5 (68.1, 82.1)	75.5 (77.2, 80.3)	62.8 (63.6, 69.9)	58.7 (61.7, 82.9)	69.8 (77.7, 80.3)
Spleen	CT	92.6 (90.3, 95.1)	95.3 (93.7, 97.1)	96.4 (95.3, 97.4)	86.0 (81.2, 92.8)	96.8 (90.8, 99.2)	98.5 (96.4, 99.6)
Stomach	CT	86.0 (82.0, 88.7)	93.0 (90.9, 95.0)	95.0 (93.5, 96.4)	71.1 (62.1, 79.9)	89.2 (80.9, 95.1)	94.2 (89.8, 97.5)
Throat Cancer	CT	74.3 (76.6, 88.2)	72.7 (73.0, 87.8)	84.0 (87.6, 94.3)	84.9 (85.6, 95.0)	87.3 (88.7, 95.4)	92.0 (94.1, 99.8)
Aorta	MR	83.9 (82.9, 91.5)	89.9 (88.6, 91.1)	93.8 (93.6, 94.2)	79.0 (77.6, 93.9)	92.6 (90.7, 95.5)	96.8 (96.7, 98.7)
Brainstem	MR	86.6 (86.5, 89.9)	87.7 (87.6, 88.4)	95.2 (95.2, 95.9)	96.0 (95.4, 99.4)	96.7 (96.3, 98.9)	100.0 (100.0, 100.0)
Cerebellum	MR	88.3 (88.3, 90.2)	89.4 (89.2, 92.8)	95.1 (95.0, 95.6)	97.8 (97.8, 99.3)	99.0 (98.9, 99.2)	100.0 (100.0, 100.0)
Deep Grey Matter	MR	76.0 (73.4, 84.4)	82.5 (80.7, 90.7)	93.6 (93.5, 94.7)	83.1 (82.7, 85.6)	90.0 (89.3, 95.3)	98.3 (98.1, 99.9)
Esophagus	MR	66.8 (66.6, 83.7)	73.0 (72.2, 77.5)	79.0 (78.6, 94.0)	75.3 (70.9, 89.0)	82.6 (80.5, 92.6)	88.5 (87.8, 99.8)
Gallbladder	MR	78.5 (78.0, 85.8)	89.6 (88.9, 92.8)	93.8 (93.4, 94.8)	74.3 (72.1, 85.5)	92.7 (91.1, 98.0)	98.8 (98.4, 99.4)
Glioma Enhancing Tumor T2	MR	83.8 (79.0, 86.9)	84.3 (80.3, 88.4)	87.0 (83.6, 89.9)	76.9 (69.9, 82.8)	81.3 (75.1, 87.7)	86.8 (84.0, 92.5)
Glioma FLAIR	MR	90.0 (87.1, 93.1)	92.2 (89.9, 94.1)	93.0 (90.7, 94.3)	89.2 (79.6, 93.6)	94.6 (90.1, 97.1)	95.4 (91.7, 97.8)
Glioma T1	MR	83.5 (80.4, 86.6)	86.0 (82.3, 88.7)	89.1 (85.9, 91.1)	69.2 (64.0, 75.6)	79.2 (75.3, 84.4)	86.3 (82.3, 90.8)
Inferior Vena Cava	MR	75.2 (73.8, 78.7)	78.7 (75.3, 83.5)	89.4 (88.5, 92.0)	72.7 (72.1, 75.4)	80.4 (78.2, 88.3)	95.8 (94.6, 97.3)
Ischemic Stroke ADC	MR	82.3 (76.3, 88.4)	82.9 (81.3, 87.6)	91.3 (90.2, 93.0)	79.8 (78.3, 85.5)	89.0 (85.0, 91.9)	98.3 (97.5, 99.2)
Ischemic Stroke DWI	MR	90.6 (88.0, 92.9)	89.2 (88.1, 92.3)	92.2 (91.5, 93.9)	95.0 (92.0, 98.0)	95.3 (94.9, 98.3)	99.3 (98.8, 99.5)
Left Atrium	MR	87.5 (86.6, 89.4)	89.5 (89.0, 91.2)	94.8 (94.6, 95.6)	72.7 (71.0, 76.7)	80.3 (80.2, 88.9)	97.2 (97.1, 98.4)
Left Kidney	MR	92.4 (91.9, 95.2)	93.9 (93.6, 94.6)	96.2 (96.1, 96.6)	89.1 (87.3, 96.6)	96.9 (96.7, 98.5)	99.3 (99.3, 99.7)
Left Ventricle	MR	88.4 (84.6, 94.1)	93.1 (92.7, 95.9)	97.6 (96.6, 98.3)	90.1 (88.3, 94.7)	95.7 (94.3, 98.2)	99.5 (99.3, 99.9)
Liver	MR	87.9 (87.6, 91.0)	94.1 (93.5, 95.6)	97.1 (96.9, 97.7)	68.6 (63.3, 74.2)	84.9 (80.9, 91.3)	96.3 (95.5, 98.1)
Meningioma T1-CE	MR	92.9 (92.8, 95.7)	95.0 (94.9, 97.1)	95.4 (95.1, 97.6)	94.2 (94.2, 99.7)	99.0 (98.8, 99.7)	99.6 (99.6, 99.9)
Meningiomai T2-FLAIR	MR	89.6 (89.0, 92.1)	90.3 (89.3, 93.4)	93.0 (92.9, 95.1)	81.0 (80.5, 90.5)	85.0 (84.8, 92.4)	94.9 (94.5, 97.3)
Prostate	MR	91.6 (90.8, 92.5)	93.9 (93.4, 94.5)	97.2 (97.0, 97.4)	94.8 (93.6, 97.1)	98.7 (98.0, 99.3)	99.8 (99.7, 99.9)
Prostate ADC	MR	91.1 (89.3, 92.0)	92.8 (90.3, 94.0)	95.7 (95.2, 96.7)	92.4 (89.6, 94.4)	96.0 (92.3, 97.3)	98.5 (98.2, 99.1)
Right Kidney	MR	90.6 (87.9, 93.0)	93.2 (92.9, 94.6)	95.6 (95.5, 96.4)	79.3 (76.9, 91.8)	95.8 (95.5, 96.7)	99.0 (98.8, 99.5)
Right Ventricle	MR	87.4 (85.8, 92.7)	93.5 (93.0, 95.2)	96.7 (96.5, 97.8)	86.3 (84.6, 90.4)	96.6 (92.1, 98.5)	99.6 (99.4, 99.8)
Spine	MR	69.9 (69.7, 72.3)	87.4 (87.2, 89.2)	91.4 (90.7, 92.3)	84.6 (84.3, 85.8)	96.8 (96.6, 97.6)	98.7 (98.6, 98.9)
Spleen	MR	92.3 (91.7, 94.2)	93.4 (93.0, 94.8)	95.9 (95.7, 96.5)	86.4 (85.3, 95.3)	94.6 (93.8, 95.4)	98.3 (97.9, 99.0)
Stomach	MR	78.3 (77.2, 82.9)	86.9 (85.8, 89.8)	94.1 (93.4, 95.1)	67.3 (66.3, 75.2)	83.2 (81.9, 86.9)	95.8 (95.1, 98.3)
Stroke T1	MR	79.8 (77.2, 82.8)	80.8 (77.4, 84.2)	82.8 (81.4, 85.3)	94.0 (90.3, 96.2)	95.0 (91.7, 97.0)	96.7 (94.1, 98.7)
Ventricles	MR	60.0 (59.2, 71.6)	73.2 (72.7, 80.8)	89.3 (89.2, 92.4)	72.6 (71.5, 78.2)	80.1 (79.9, 83.6)	89.9 (89.5, 95.0)
Vestibular Schwannoma	MR	90.8 (89.8, 93.2)	93.1 (92.6, 95.6)	96.5 (96.0, 97.6)	99.3 (98.9, 99.8)	99.9 (99.6, 100.0)	100.0 (100.0, 100.0)

TABLE 13
Performance change with varying training data size on the internal validation set (Chest X-Ray, Dermoscopy, Endoscopy Fundus, Mammography, OCT, Pathology, and Ultrasound images) in terms of DSC and NSD scores. Scores for each task are summarized as Median values (First quartile, Third quartile). Source data are provided as a Source Data file.

Task	Modality	DSC			NSD		
		10K	100K	1M	10K	100K	1M
COVID-19 CXR	CXR	86.8 (81.4, 91.5)	88.9 (83.3, 93.9)	92.3 (88.7, 95.3)	94.2 (90.5, 96.9)	95.7 (91.9, 98.3)	98.1 (96.4, 99.3)
Heart CXR	CXR	93.4 (92.8, 94.3)	96.3 (95.4, 97.4)	97.2 (96.7, 98.0)	96.8 (96.5, 97.9)	98.9 (98.4, 99.6)	99.5 (99.2, 99.8)
Left Lung CXR	CXR	97.3 (96.2, 98.0)	98.1 (97.6, 98.6)	98.6 (98.1, 98.9)	99.7 (99.2, 99.9)	99.9 (99.8, 100.0)	100.0 (99.9, 100.0)
Pneumonia CXR	CXR	94.8 (92.7, 96.3)	96.8 (95.7, 97.7)	97.4 (96.4, 98.1)	98.5 (96.8, 99.3)	99.6 (98.8, 99.9)	99.8 (99.4, 100.0)
Pneumothorax CXR	CXR	74.8 (66.9, 80.5)	76.3 (70.1, 83.2)	94.4 (93.7, 95.2)	80.5 (72.5, 85.8)	82.1 (76.5, 88.5)	98.2 (97.6, 98.7)
Right Lung CXR	CXR	96.9 (95.6, 97.7)	98.0 (97.3, 98.5)	98.4 (97.8, 98.8)	99.6 (98.8, 99.8)	100.0 (99.7, 100.0)	100.0 (99.9, 100.0)
Skin Cancer Dermoscopy	Dermoscopy	93.6 (91.0, 95.5)	95.3 (93.2, 96.7)	96.4 (95.0, 97.7)	98.1 (96.2, 99.2)	99.1 (97.7, 99.8)	99.7 (98.9, 99.9)
Cholecystectomy Endoscopy	Endoscopy	54.0 (43.1, 64.2)	73.0 (63.0, 84.5)	87.7 (70.4, 93.5)	59.2 (47.5, 69.2)	77.8 (67.3, 88.6)	92.2 (73.9, 96.5)
Gastrectomy Endoscopy	Endoscopy	57.6 (45.0, 68.0)	79.0 (69.3, 86.1)	88.1 (79.2, 92.8)	61.7 (48.9, 72.4)	83.3 (73.8, 90.2)	92.2 (83.1, 96.4)
Polyp Endoscopy	Endoscopy	88.3 (83.0, 92.0)	94.6 (89.5, 96.4)	97.9 (96.7, 98.5)	92.4 (85.8, 96.0)	97.1 (92.4, 99.3)	99.3 (98.7, 100.0)
Surgical Instrument Endoscopy	Endoscopy	81.9 (74.8, 86.9)	90.0 (83.3, 94.5)	95.1 (93.9, 96.8)	85.2 (78.4, 89.1)	93.8 (85.4, 96.5)	97.5 (96.7, 98.3)
Glaucoma Cup Fundus	Fundus	76.2 (75.1, 82.0)	81.2 (77.3, 89.7)	96.3 (95.5, 97.2)	78.5 (77.4, 84.1)	83.1 (79.8, 92.1)	98.6 (98.5, 99.3)
Glaucoma Disc Fundus	Fundus	90.9 (89.9, 93.7)	95.7 (95.5, 96.2)	97.8 (97.7, 98.2)	92.3 (91.4, 94.9)	97.4 (97.2, 98.1)	99.2 (99.1, 99.4)
Non-Glaucoma Cup Fundus	Fundus	75.6 (72.0, 78.8)	84.4 (79.2, 89.5)	95.6 (94.9, 96.3)	78.7 (75.3, 81.7)	88.3 (83.1, 93.1)	98.7 (98.3, 99.1)
Non-Glaucoma Disc Fundus	Fundus	87.9 (86.3, 90.1)	95.4 (93.8, 96.6)	97.5 (97.2, 98.0)	89.6 (88.0, 91.9)	97.4 (95.6, 98.4)	99.2 (98.9, 99.4)
Breast Cancer Mammography	Mammography	75.0 (63.5, 83.1)	78.6 (72.8, 84.8)	83.4 (79.8, 88.5)	87.1 (72.9, 94.3)	91.2 (86.3, 95.3)	94.7 (91.7, 97.9)
Diabetic Macular Edema OCT	OCT	88.4 (85.1, 92.4)	91.3 (89.2, 94.0)	94.8 (93.2, 96.3)	97.8 (95.6, 99.9)	99.7 (98.7, 100.0)	100.0 (100.0, 100.0)
Glomeruli Pathology	Pathology	91.8 (89.1, 94.3)	95.3 (93.6, 96.4)	97.5 (97.1, 97.9)	96.7 (94.6, 98.2)	98.9 (97.9, 99.7)	99.9 (99.7, 100.0)
Intestine FTU Pathology	Pathology	85.1 (81.7, 88.5)	93.1 (91.3, 95.0)	95.9 (95.5, 96.7)	93.5 (91.3, 95.5)	99.1 (98.1, 99.8)	99.8 (99.6, 100.0)
Benign Breast Cancer US	US	91.7 (89.2, 92.9)	92.3 (90.0, 94.6)	94.1 (92.3, 95.4)	96.6 (94.2, 98.4)	98.4 (96.7, 99.1)	98.4 (97.7, 99.6)
Benign Thyroid Nodule US	US	90.7 (86.9, 93.1)	93.1 (90.6, 95.3)	95.1 (93.2, 96.9)	95.3 (93.2, 96.9)	97.4 (95.7, 98.8)	98.7 (97.7, 99.5)
Fetal Head US	US	95.0 (94.2, 95.6)	96.8 (96.0, 97.5)	98.3 (97.7, 98.8)	98.2 (97.5, 98.7)	99.3 (98.8, 99.7)	100.0 (99.8, 100.0)
Kidney US	US	94.8 (92.5, 96.2)	97.5 (96.5, 98.2)	97.8 (96.9, 98.4)	98.0 (96.1, 98.9)	99.7 (99.2, 99.9)	99.8 (99.6, 99.9)
Left Atrium US	US	93.2 (91.4, 94.5)	94.9 (93.2, 96.0)	98.3 (97.9, 98.6)	97.6 (96.2, 98.4)	98.7 (97.6, 99.4)	100.0 (100.0, 100.0)
Left Ventricle US	US	92.4 (90.6, 93.8)	94.0 (92.0, 95.4)	98.3 (97.8, 98.6)	97.3 (96.0, 98.3)	98.4 (97.1, 99.2)	100.0 (100.0, 100.0)
Malignant Breast Cancer US	US	87.0 (85.0, 91.5)	91.6 (88.8, 94.3)	94.0 (93.0, 96.9)	91.7 (90.7, 93.7)	95.0 (94.1, 97.6)	98.5 (96.6, 98.9)
Malignant Thyroid Nodule US	US	88.2 (85.2, 90.6)	91.6 (88.0, 93.6)	93.2 (91.4, 95.1)	95.0 (93.1, 97.1)	97.9 (96.1, 98.8)	98.9 (97.6, 99.6)
Nerve Cancer US	US	86.5 (82.8, 89.5)	87.5 (84.1, 89.8)	90.9 (88.5, 92.3)	94.6 (90.8, 96.6)	95.4 (92.2, 97.1)	97.6 (96.3, 98.6)
Pubic Symphysis US	US	89.6 (88.7, 91.3)	92.0 (91.8, 93.1)	93.5 (93.0, 95.5)	97.2 (96.6, 98.4)	98.9 (98.8, 99.4)	99.5 (99.2, 100.0)

TABLE 14
Performance change with varying training data size on the external validation set in terms of DSC and NSD scores. Scores for each task are summarized as Median values (First quartile, Third quartile). Source data are provided as a Source Data file.

Task	Modality	DSC			NSD		
		10K	100K	1M	10K	100K	1M
Adrenocortical carcinoma	CT	89.6 (87.4, 91.4)	91.7 (90.4, 94.1)	92.7 (91.2, 94.7)	85.1 (72.7, 90.3)	92.2 (85.4, 96.9)	93.8 (88.9, 97.5)
Aorta	CT	91.4 (88.9, 92.6)	93.9 (92.5, 94.6)	94.3 (93.6, 94.8)	92.8 (88.7, 95.2)	97.1 (93.9, 98.4)	98.4 (97.8, 98.9)
Brainstem	CT	70.0 (67.9, 72.6)	59.4 (56.1, 62.2)	77.5 (74.5, 81.5)	53.8 (46.5, 65.1)	40.0 (36.6, 48.3)	71.8 (64.3, 76.7)
Esophagus	CT	72.7 (62.1, 79.1)	76.8 (62.8, 81.8)	78.0 (67.9, 83.1)	82.3 (68.5, 89.7)	88.5 (71.3, 93.5)	89.2 (76.3, 93.8)
Gallbladder	CT	74.0 (67.9, 80.9)	83.1 (77.8, 87.1)	86.6 (78.7, 90.2)	78.5 (72.0, 83.4)	92.2 (86.0, 96.6)	95.0 (87.8, 97.8)
Heart	CT	90.7 (89.4, 91.6)	92.0 (89.9, 93.3)	96.4 (96.3, 96.6)	76.7 (71.7, 82.0)	82.8 (74.8, 86.5)	96.4 (96.0, 97.5)
Hepatocellular carcinoma	CT	85.7 (81.1, 89.6)	87.2 (84.1, 91.0)	95.0 (93.4, 96.3)	76.3 (69.7, 82.1)	82.7 (79.2, 88.3)	96.9 (95.5, 98.2)
Inferior Vena Cava	CT	73.1 (69.0, 76.7)	83.8 (79.4, 86.1)	84.8 (81.2, 87.5)	63.1 (58.2, 67.8)	82.2 (79.0, 86.2)	85.7 (81.8, 89.4)
Left Adrenal Gland	CT	58.5 (51.8, 64.5)	68.3 (60.8, 72.8)	70.0 (62.5, 74.4)	72.9 (66.5, 78.4)	83.8 (75.8, 89.1)	85.2 (75.8, 90.3)
Left Kidney	CT	91.2 (89.4, 92.4)	93.5 (92.6, 94.3)	95.6 (95.0, 95.9)	87.2 (82.9, 90.6)	94.0 (92.5, 95.5)	98.6 (97.8, 99.0)
Left Lung	CT	93.1 (92.1, 94.6)	92.7 (91.5, 94.1)	96.2 (95.8, 96.9)	85.3 (81.4, 89.7)	82.8 (77.4, 88.3)	96.1 (94.9, 96.9)
Liver	CT	92.2 (90.2, 93.3)	94.5 (93.4, 95.3)	96.7 (96.2, 97.1)	79.8 (74.9, 84.4)	90.7 (86.9, 93.4)	96.3 (94.7, 97.4)
Lung tumor	CT	66.6 (59.7, 76.5)	58.6 (55.6, 68.4)	74.8 (61.6, 88.7)	72.3 (66.3, 81.9)	61.8 (57.2, 69.1)	77.1 (68.1, 90.1)
Lymph node	CT	73.6 (68.8, 78.9)	77.8 (71.3, 82.6)	81.2 (75.7, 85.2)	82.2 (76.9, 86.8)	84.5 (77.3, 90.9)	90.5 (84.3, 95.2)
Nasopharynx cancer	CT	68.6 (65.5, 73.2)	64.9 (62.1, 68.2)	87.8 (85.0, 91.4)	62.1 (57.6, 68.2)	69.2 (65.5, 74.9)	92.0 (87.7, 95.3)
Pancreas	CT	68.9 (61.4, 74.3)	79.2 (76.2, 82.8)	87.0 (83.8, 89.2)	73.7 (67.6, 79.8)	86.4 (83.0, 90.3)	93.8 (89.5, 96.5)
Parotid L	CT	65.5 (62.3, 71.3)	62.2 (58.5, 67.0)	83.1 (80.2, 87.0)	56.9 (51.2, 61.8)	55.5 (51.7, 61.0)	83.7 (76.2, 89.6)
Parotid R	CT	66.0 (62.8, 69.5)	61.4 (59.3, 65.6)	81.2 (77.7, 86.9)	54.5 (51.3, 61.2)	55.7 (51.8, 61.6)	82.0 (75.1, 88.7)
Right Adrenal Gland	CT	53.6 (47.4, 61.5)	64.5 (57.2, 70.7)	70.8 (64.8, 75.5)	68.7 (61.8, 76.4)	83.3 (77.1, 89.8)	88.7 (82.1, 93.7)
Right Kidney	CT	91.3 (89.5, 92.9)	93.2 (92.7, 93.9)	95.3 (95.0, 95.7)	88.0 (82.8, 91.3)	93.1 (91.2, 94.6)	98.5 (98.2, 98.9)
Right Lung	CT	94.9 (94.0, 95.8)	94.2 (93.4, 95.0)	96.8 (96.6, 97.3)	88.1 (83.0, 91.7)	85.1 (78.2, 89.0)	96.3 (95.5, 97.1)
Spleen	CT	88.5 (86.9, 90.0)	94.1 (93.1, 94.9)	95.7 (95.3, 96.1)	81.0 (77.8, 85.1)	97.4 (95.3, 98.3)	98.8 (98.3, 99.2)
Stomach	CT	85.5 (81.9, 88.3)	92.5 (90.7, 94.0)	95.3 (94.5, 96.0)	72.9 (67.0, 78.9)	91.3 (86.8, 94.5)	97.7 (95.6, 98.5)
Brain lesion	MR	83.5 (80.2, 88.3)	82.0 (78.5, 88.7)	96.3 (95.3, 97.4)	90.3 (85.9, 96.3)	88.2 (85.3, 96.4)	100.0 (100.0, 100.0)
Brainstem	MR	85.2 (79.3, 88.4)	87.3 (84.5, 89.5)	95.1 (94.8, 95.7)	88.6 (69.7, 94.2)	88.8 (80.5, 94.4)	99.5 (98.4, 99.9)
Cervical cancer	MR	75.2 (77.9, 87.2)	81.1 (82.4, 87.0)	93.5 (93.6, 93.9)	83.0 (83.1, 95.4)	92.7 (93.0, 95.5)	99.6 (99.6, 100.0)
Eye L	MR	85.0 (83.0, 89.3)	83.8 (82.4, 85.9)	96.0 (95.6, 96.9)	89.5 (83.4, 93.9)	86.4 (82.6, 90.7)	100.0 (100.0, 100.0)
Eye R	MR	86.3 (82.2, 88.8)	83.8 (82.3, 85.7)	96.2 (95.5, 96.7)	89.0 (82.6, 95.1)	84.4 (81.1, 90.0)	100.0 (100.0, 100.0)
Glottis	MR	35.6 (21.8, 44.3)	48.0 (42.6, 54.3)	81.6 (77.0, 86.8)	69.7 (61.0, 72.4)	72.8 (69.9, 80.0)	98.2 (96.0, 99.4)
Left Kidney T1-Inphase	MR	91.2 (87.9, 92.8)	93.0 (91.6, 94.3)	96.1 (95.8, 97.3)	96.8 (95.9, 98.8)	98.8 (96.9, 99.6)	100.0 (100.0, 100.0)
Left Kidney T1-Outphase	MR	86.5 (84.6, 89.1)	91.3 (90.3, 92.8)	96.3 (95.8, 97.2)	94.1 (93.7, 96.6)	97.5 (95.5, 98.9)	100.0 (100.0, 100.0)
Left Kidney T2	MR	92.2 (90.9, 94.6)	92.8 (91.4, 94.4)	97.1 (96.1, 97.6)	98.3 (96.5, 99.0)	96.2 (95.2, 99.3)	99.9 (99.8, 100.0)
Left ventricle cavity	MR	89.2 (85.8, 92.4)	91.0 (87.2, 93.6)	96.6 (95.7, 97.2)	91.1 (86.3, 94.8)	94.0 (89.1, 96.6)	99.4 (99.1, 99.7)
Lips	MR	53.0 (48.3, 60.6)	44.3 (39.8, 54.7)	86.3 (84.0, 88.6)	68.4 (64.0, 72.7)	72.7 (69.5, 81.4)	98.4 (97.8, 99.2)
Liver T1-Inphase	MR	91.2 (90.8, 92.0)	92.8 (92.4, 93.9)	96.9 (96.4, 97.1)	91.2 (88.7, 93.1)	94.4 (93.4, 97.5)	99.7 (99.2, 99.8)
Liver T1-Outphase	MR	92.2 (91.5, 93.6)	93.8 (92.4, 94.3)	96.5 (96.2, 97.6)	94.4 (93.4, 97.6)	96.3 (95.4, 98.7)	99.5 (99.0, 99.9)
Liver T2	MR	91.0 (88.9, 91.7)	92.5 (90.7, 94.1)	97.1 (96.6, 97.5)	89.8 (87.9, 91.8)	94.2 (90.9, 97.1)	99.6 (99.4, 99.9)
Mandible Bone	MR	23.7 (22.1, 28.2)	18.4 (16.2, 23.5)	73.9 (70.8, 78.8)	41.8 (37.7, 45.5)	40.9 (37.9, 44.6)	63.7 (62.0, 70.8)
Parotid L	MR	71.7 (68.2, 76.5)	81.7 (78.3, 83.8)	90.8 (90.4, 92.1)	72.1 (68.5, 79.3)	86.7 (82.8, 92.0)	98.5 (97.9, 99.0)
Parotid R	MR	75.6 (71.9, 77.0)	80.1 (77.0, 83.2)	90.8 (90.1, 91.5)	77.1 (72.8, 82.2)	85.2 (81.8, 90.5)	98.4 (97.7, 98.9)
Prostate	MR	90.9 (88.1, 93.4)	92.5 (90.9, 95.0)	97.4 (97.2, 97.9)	93.4 (91.2, 95.2)	95.0 (93.3, 97.2)	99.4 (99.2, 99.7)
Right Kidney T1-Inphase	MR	88.2 (86.0, 92.5)	92.7 (91.1, 93.9)	95.9 (94.7, 97.2)	96.0 (91.9, 97.3)	98.6 (96.9, 99.8)	100.0 (99.9, 100.0)
Right Kidney T1-Outphase	MR	85.6 (83.5, 89.3)	91.3 (90.1, 93.5)	96.3 (95.4, 97.2)	94.6 (89.4, 98.1)	97.7 (96.4, 99.5)	100.0 (100.0, 100.0)
Right Kidney T2	MR	93.8 (93.4, 94.9)	93.9 (92.7, 94.5)	96.9 (96.2, 97.9)	98.9 (97.5, 99.6)	97.3 (96.5, 99.4)	100.0 (99.9, 100.0)
Right ventricle cavity	MR	84.2 (78.6, 89.0)	89.8 (85.2, 92.8)	95.4 (94.5, 96.2)	87.3 (82.3, 91.5)	94.2 (90.0, 97.0)	99.2 (98.9, 99.5)
Spinal Cord	MR	60.6 (58.2, 63.2)	54.0 (50.9, 57.6)	70.4 (67.9, 75.5)	70.6 (64.6, 75.8)	56.9 (51.4, 61.4)	81.6 (78.7, 85.7)
Spleen T1-Inphase	MR	81.8 (80.2, 85.2)	90.4 (89.6, 92.6)	95.6 (94.9, 96.2)	82.8 (81.8, 89.1)	96.8 (95.0, 99.1)	100.0 (99.9, 100.0)
Spleen T1-Outphase	MR	85.7 (84.0, 87.5)	90.2 (88.8, 92.5)	95.2 (94.9, 96.7)	91.6 (87.3, 95.0)	98.2 (97.3, 99.6)	99.9 (99.8, 100.0)
Spleen T2	MR	87.9 (85.6, 90.8)	94.8 (93.9, 95.8)	97.0 (96.2, 97.9)	88.5 (86.3, 95.6)	99.7 (99.4, 100.0)	100.0 (100.0, 100.0)
Supraglottic Larynx	MR	56.2 (47.7, 62.3)	62.6 (57.1, 69.1)	86.3 (84.4, 88.9)	80.6 (78.3, 86.4)	85.5 (79.2, 90.6)	97.9 (97.2, 99.3)
COVID-19 Lung CXR	CXR	96.1 (94.9, 97.1)	98.1 (97.3, 98.6)	97.9 (97.3, 98.3)	99.2 (98.4, 99.7)	99.9 (99.7, 100.0)	99.9 (99.8, 100.0)
Lung Opacity CXR	CXR	95.1 (93.0, 96.4)	97.3 (96.2, 98.1)	97.5 (96.7, 98.1)	98.6 (97.2, 99.3)	99.7 (99.1, 99.9)	99.8 (99.5, 100.0)
Normal Lung CXR	CXR	97.6 (97.0, 97.9)	98.9 (98.6, 99.1)	98.7 (98.3, 98.9)	99.8 (99.5, 99.9)	100.0 (100.0, 100.0)	100.0 (100.0, 100.0)
Pneumonia Lung CXR	CXR	95.2 (92.8, 96.2)	97.6 (96.3, 98.2)	97.6 (96.5, 98.1)	99.2 (97.6, 99.6)	99.8 (99.2, 100.0)	99.9 (99.5, 100.0)
Skin Cancer Dermoscopy	Dermoscopy	90.8 (89.8, 94.1)	95.1 (94.1, 97.7)	96.5 (95.7, 97.0)	97.9 (96.9, 99.0)	99.6 (99.2, 99.9)	99.9 (99.7, 100.0)
Polyp Endoscopy	Endoscopy	91.5 (87.8, 94.0)	96.3 (93.9, 97.6)	97.7 (96.9, 98.2)	93.7 (90.6, 96.1)	98.3 (96.5, 99.3)	99.4 (99.0, 99.7)
Optic Cup Fundus	Fundus	74.7 (66.3, 80.3)	81.7 (72.4, 87.2)	95.0 (92.8, 96.0)	76.8 (68.9, 82.0)	84.2 (75.5, 89.6)	97.5 (95.8, 98.1)
Optic Disc Fundus	Fundus	90.4 (88.9, 91.7)	96.0 (94.3, 96.9)	97.7 (97.3, 98.0)	91.3 (89.8, 92.5)	97.0 (95.3, 97.8)	98.6 (98.3, 98.9)
Colon Gland Pathology	Pathology	82.2 (77.1, 86.4)	91.8 (87.1, 94.0)	95.6 (94.6, 96.3)	85.7 (81.2, 89.5)	94.8 (91.0, 96.7)	98.2 (97.4, 98.7)
Fetal Head US	US	94.4 (93.3, 95.5)	97.0 (96.1, 97.5)	97.3 (96.6, 97.9)	95.6 (94.4, 96.6)	98.1 (97.4, 98.6)	98.4 (97.7, 98.8)

TABLE 15
Annotation time in second for two experts with and without MedSAM assistance. The Manual column represents the total time taken to annotate each case without MedSAM assistance. The Initial marker column illustrates the time for generating the initial marker every 3-10 slices to prompt MedSAM. The total time taken for MedSAM to perform inference on each case is shown in the MedSAM inference column. The refinement column shows the time taken for each expert to manually revised the segmentation results until they were satisfied. The annotation time with MedSAM assistance can be calculated by summing the Initial marker, MedSAM inference, and Refinement columns. Source data are provided as a Source Data file.

Case	Expert 1				Expert 2			
	Manual	Initial marker	MedSAM inference	Refinement	Manual	Initial marker	MedSAM inference	Refinement
010	2279.0	150.0	31.04	790.0	2533.0	101.0	32.1	438.0
015	1030.0	92.0	10.31	338.0	958.0	58.0	10.58	103.0
020	1483.0	100.0	10.33	60.0	1454.0	75.0	10.74	220.0
027	1556.0	70.0	10.52	43.0	1386.0	82.0	11.33	234.0
029	2188.0	103.0	8.47	281.0	1910.0	88.0	9.56	269.0
039	754.0	90.0	4.19	121.0	680.0	55.0	4.51	114.0
040	3716.0	236.0	11.41	745.0	3546.0	198.0	14.21	369.0
042	3664.0	105.0	13.4	46.0	3431.0	120.0	17.13	173.0
043	2673.0	97.0	10.94	149.0	2416.0	117.0	12.43	370.0
050	3034.0	132.0	11.04	75.0	3136.0	80.0	12.2	259.0
Average	2237.7	117.5	12.2	264.8	2145	97.4	13.5	254.9

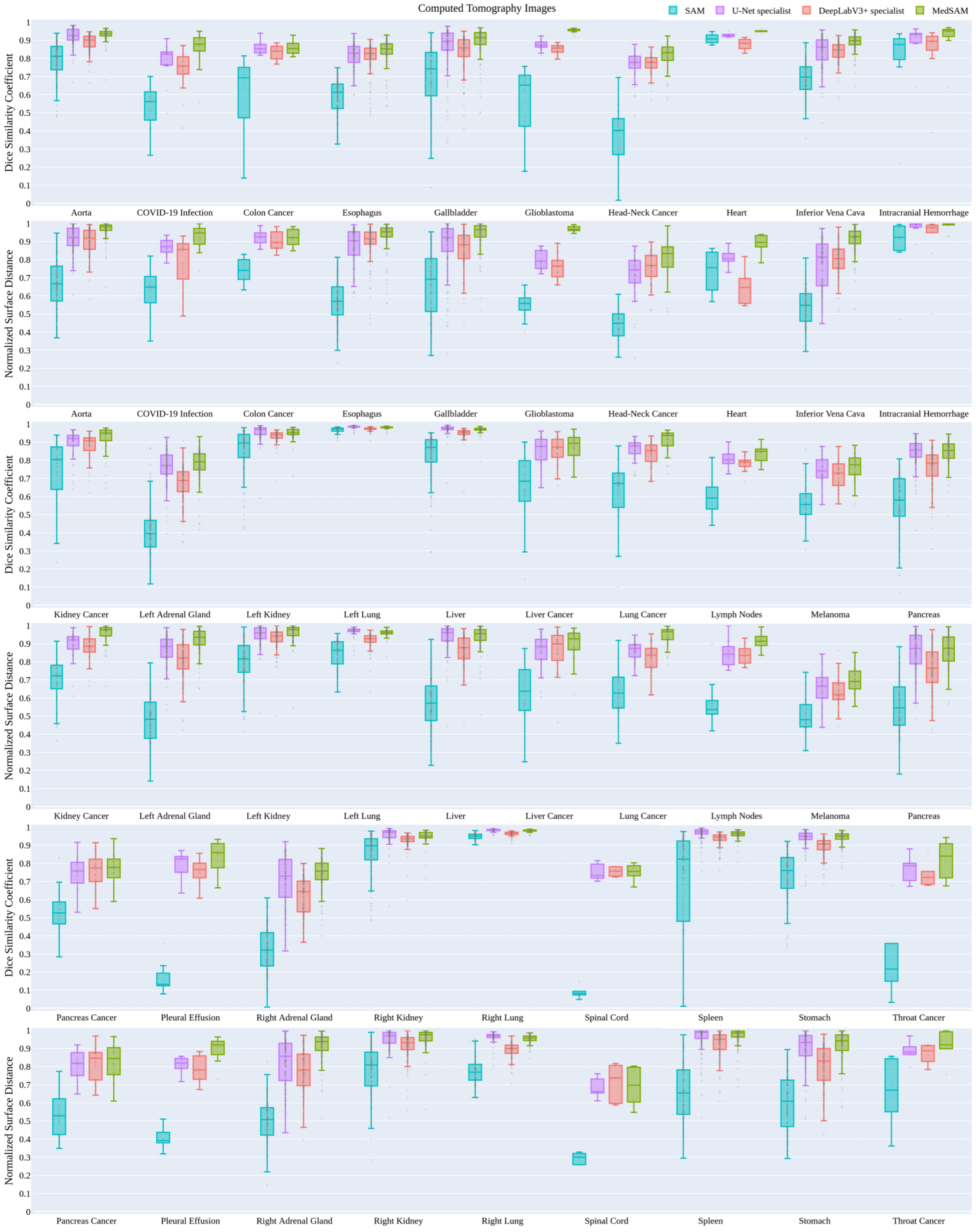

Fig. 2. Box plots of dice similarity coefficient and normalized surface distance scores for each CT segmentation task in internal validation. The center line within the box represents the median value, with the bottom and top bounds of the box delineating the 25th and 75th percentiles, respectively. Whiskers are chosen to show the 1.5 of the interquartile range. Source data are provided as a Source Data file.

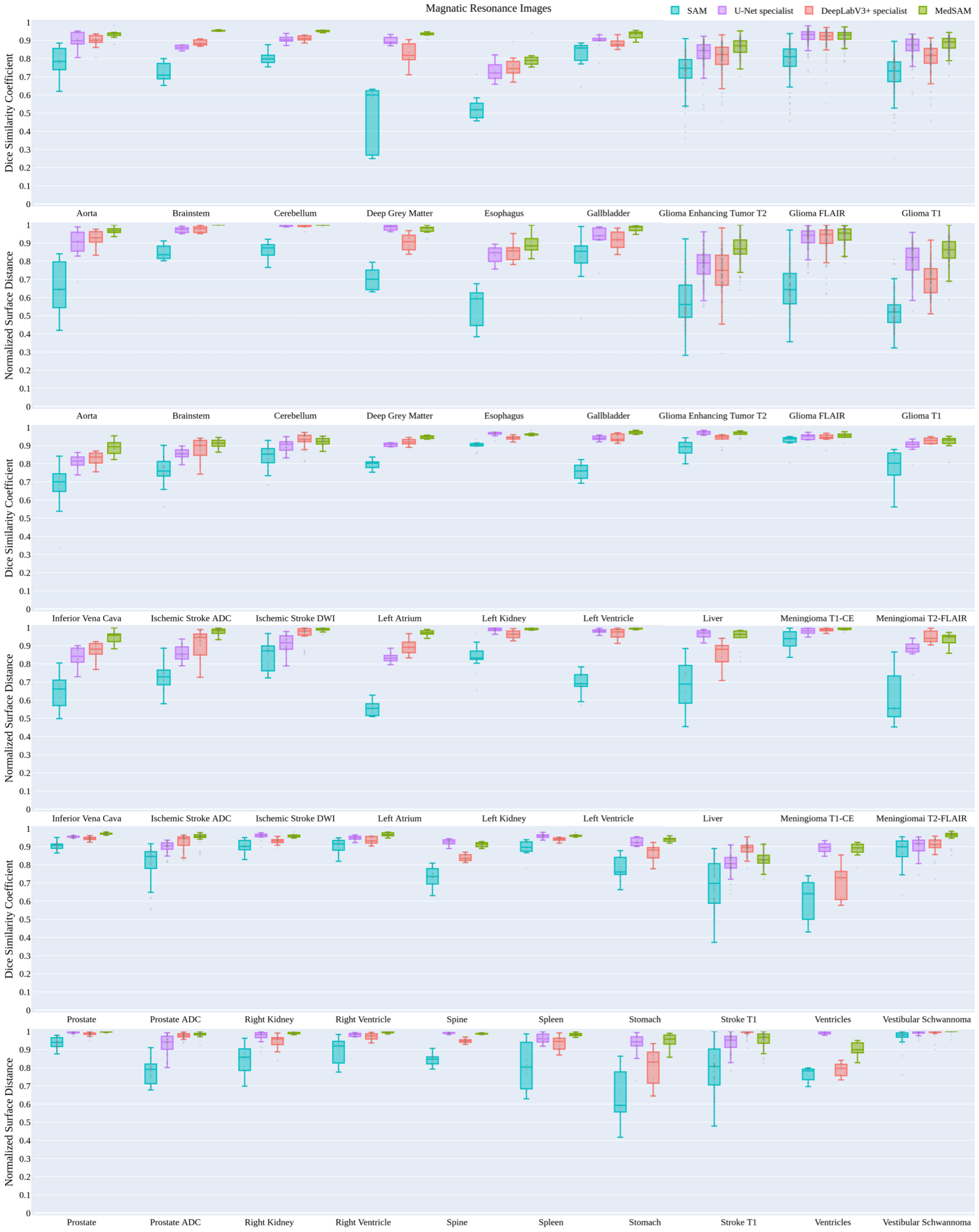

Fig. 3. Box plots of dice similarity coefficient and normalized surface distance scores for each MR segmentation task in internal validation. The center line within the box represents the median value, with the bottom and top bounds of the box delineating the 25th and 75th percentiles, respectively. Whiskers are chosen to show the 1.5 of the interquartile range. Source data are provided as a Source Data file.

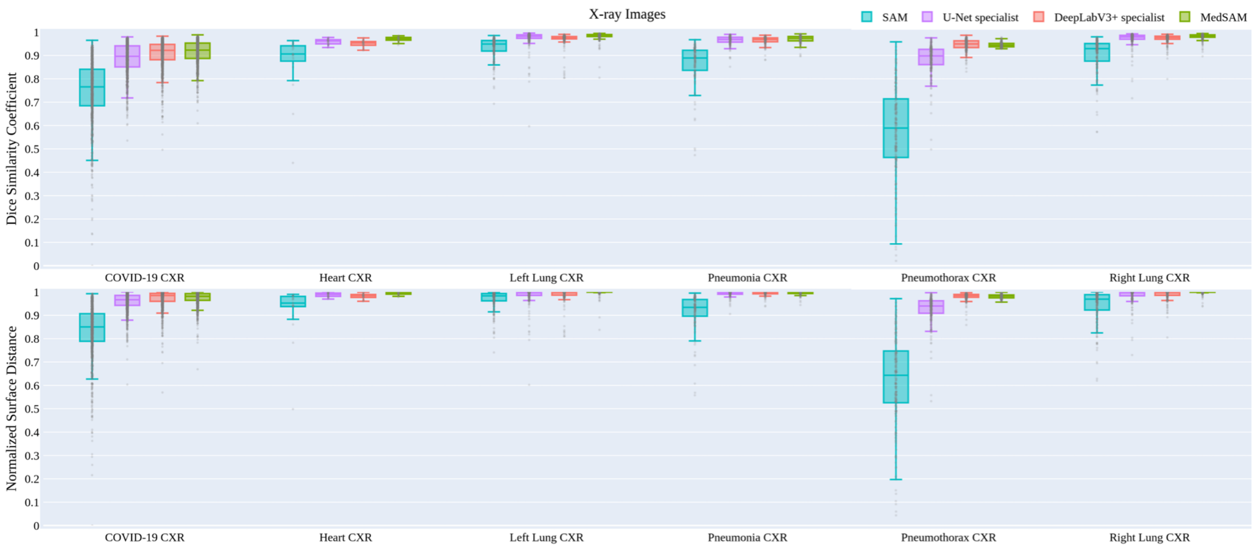

Fig. 4. Box plots of dice similarity coefficient and normalized surface distance scores for each X-Ray segmentation task in internal validation. The center line within the box represents the median value, with the bottom and top bounds of the box delineating the 25th and 75th percentiles, respectively. Whiskers are chosen to show the 1.5 of the interquartile range. Source data are provided as a Source Data file.

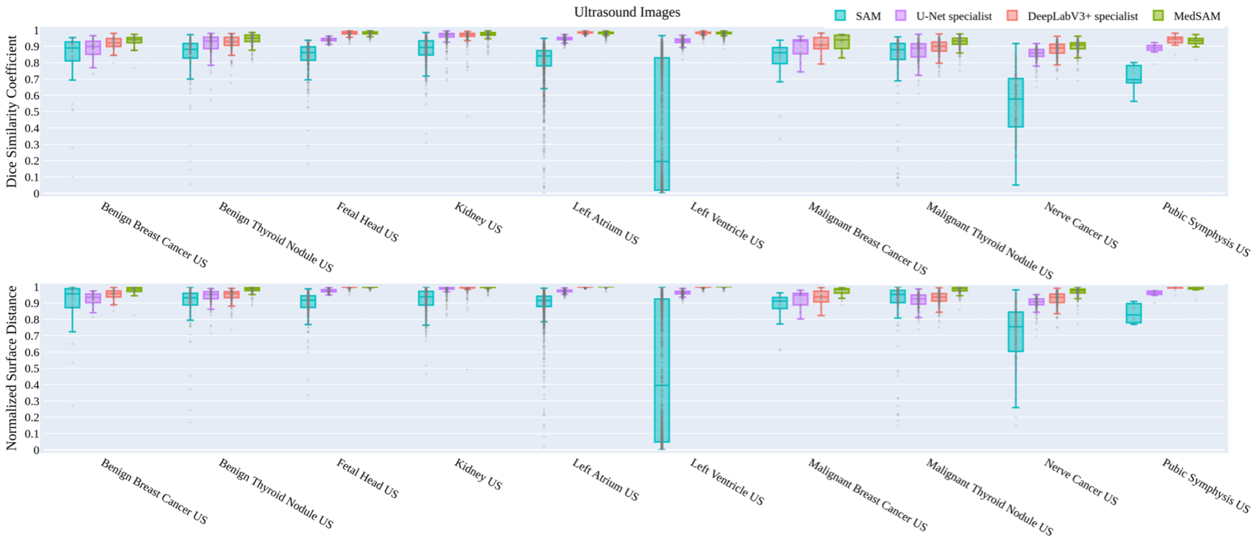

Fig. 5. Box plots of dice similarity coefficient and normalized surface distance scores for each ultrasound segmentation task in internal validation. The center line within the box represents the median value, with the bottom and top bounds of the box delineating the 25th and 75th percentiles, respectively. Whiskers are chosen to show the 1.5 of the interquartile range. Source data are provided as a Source Data file.

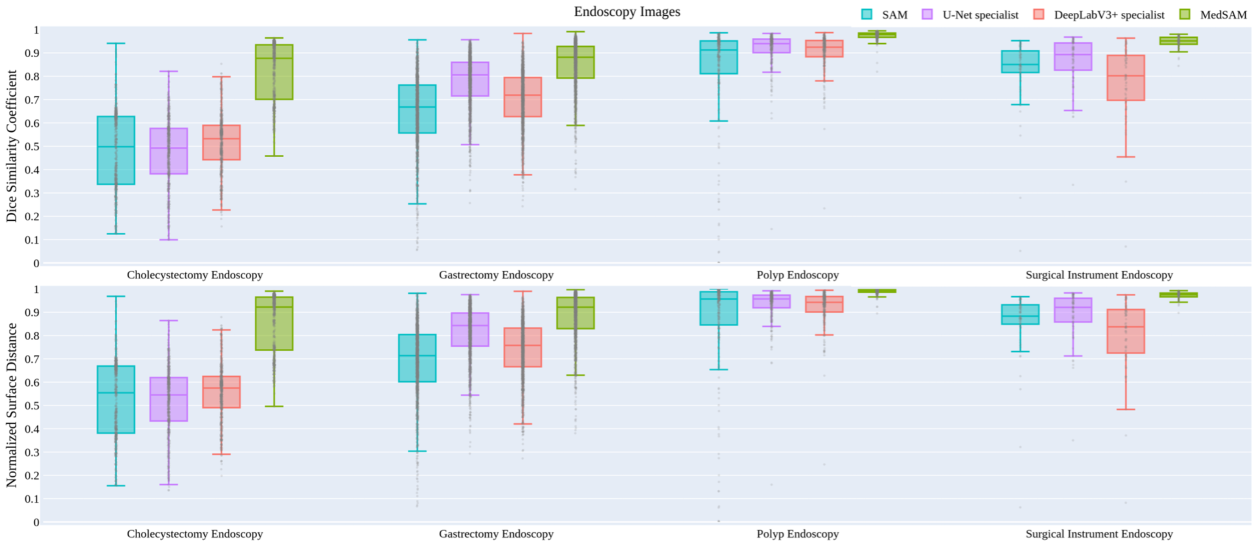

Fig. 6. Box plots of dice similarity coefficient and normalized surface distance scores for each endoscopy segmentation task in internal validation. The center line within the box represents the median value, with the bottom and top bounds of the box delineating the 25th and 75th percentiles, respectively. Whiskers are chosen to show the 1.5 of the interquartile range. Source data are provided as a Source Data file.

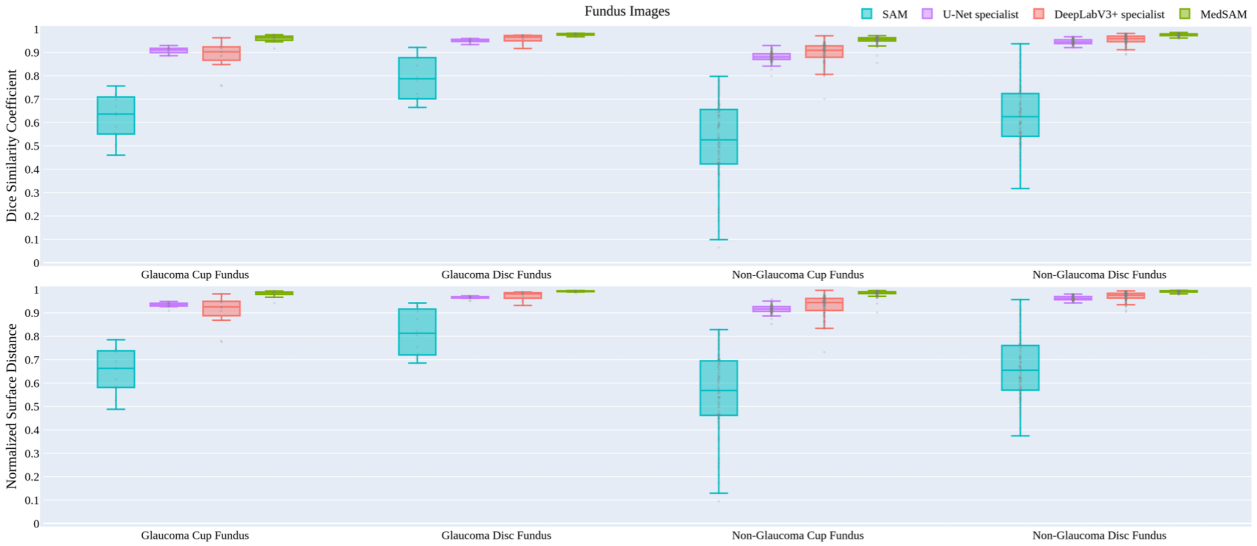

Fig. 7. Box plots of dice similarity coefficient and normalized surface distance scores for each fundus segmentation task in internal validation. The center line within the box represents the median value, with the bottom and top bounds of the box delineating the 25th and 75th percentiles, respectively. Whiskers are chosen to show the 1.5 of the interquartile range. Source data are provided as a Source Data file.

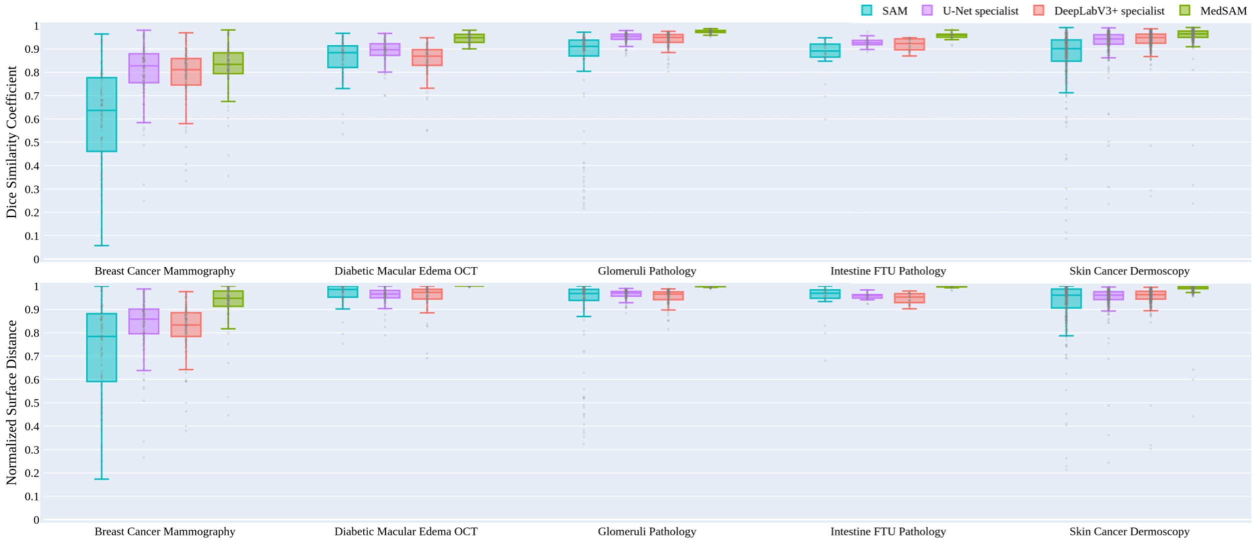

Fig. 8. Box plots of dice similarity coefficient and normalized surface distance scores for segmentation tasks of other 2D modalities in internal validation. The center line within the box represents the median value, with the bottom and top bounds of the box delineating the 25th and 75th percentiles, respectively. Whiskers are chosen to show the 1.5 of the interquartile range. Source data are provided as a Source Data file.

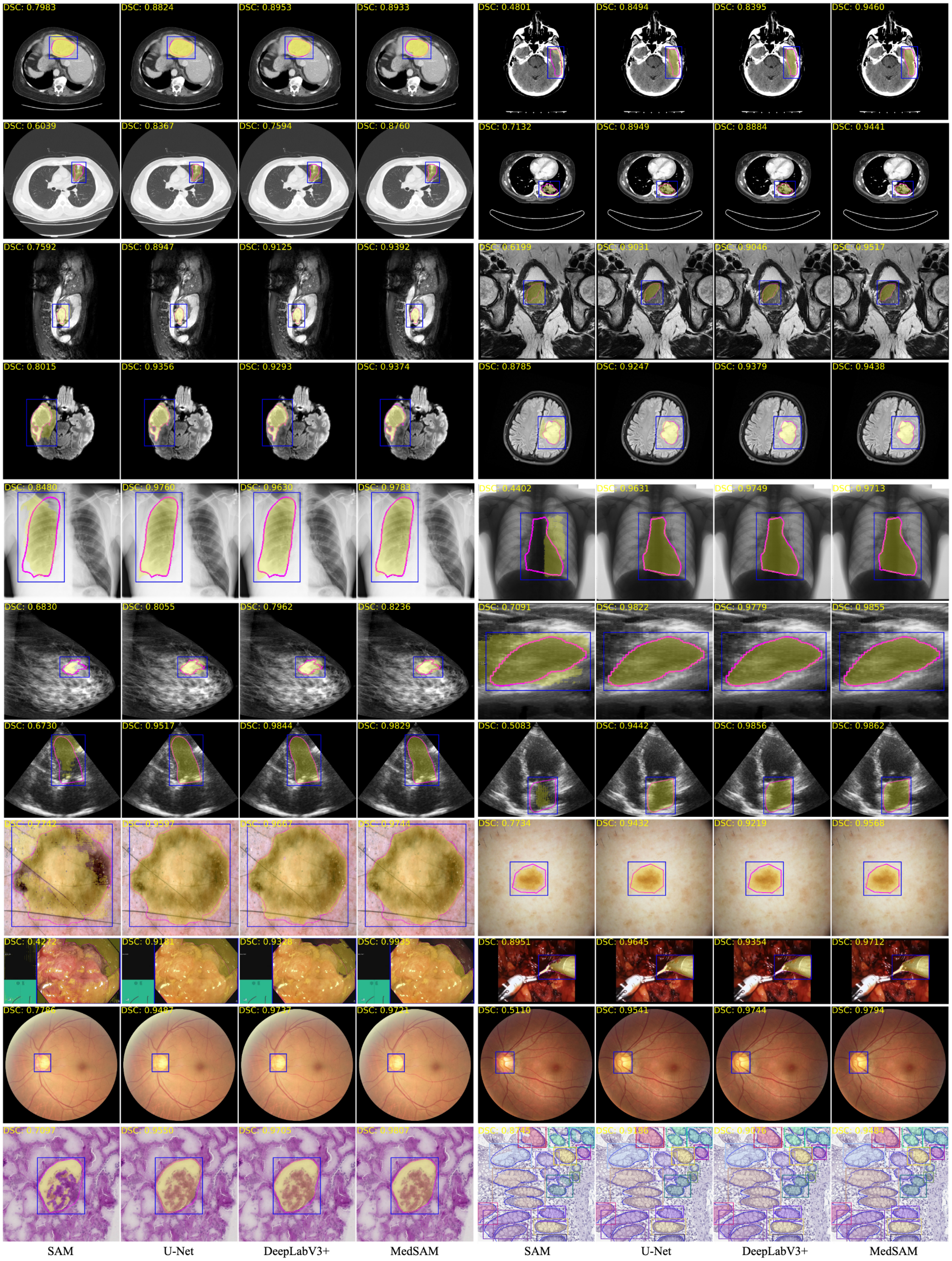

Fig. 9. Visualized segmentation examples in internal validation. The visualized examples were randomly selected where MedSAM achieved a median DSC score for the corresponding task.

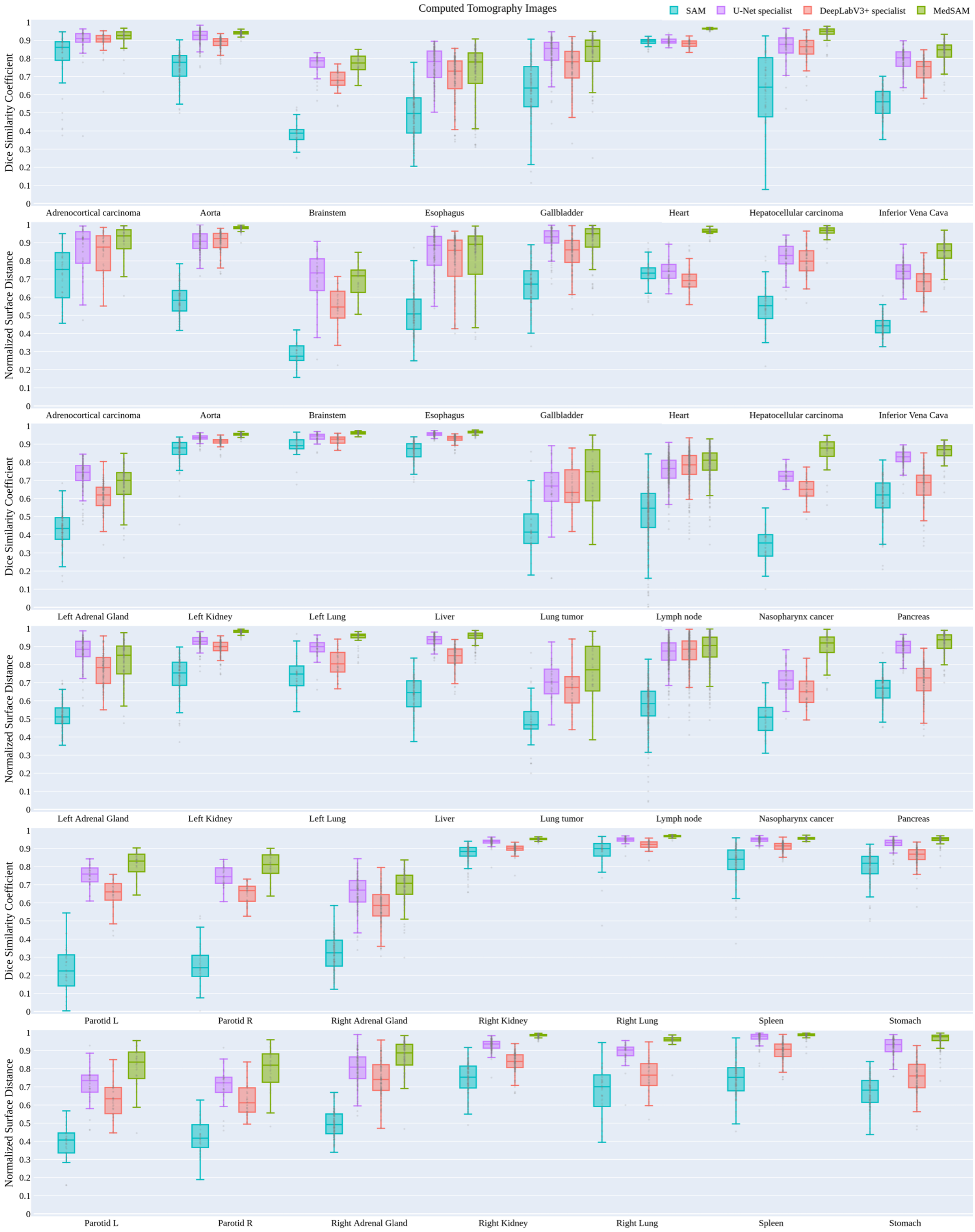

Fig. 10. Box plots of dice similarity coefficient and normalized surface distance scores for each CT segmentation task in external validation. The center line within the box represents the median value, with the bottom and top bounds of the box delineating the 25th and 75th percentiles, respectively. Whiskers are chosen to show the 1.5 of the interquartile range. Source data are provided as a Source Data file.

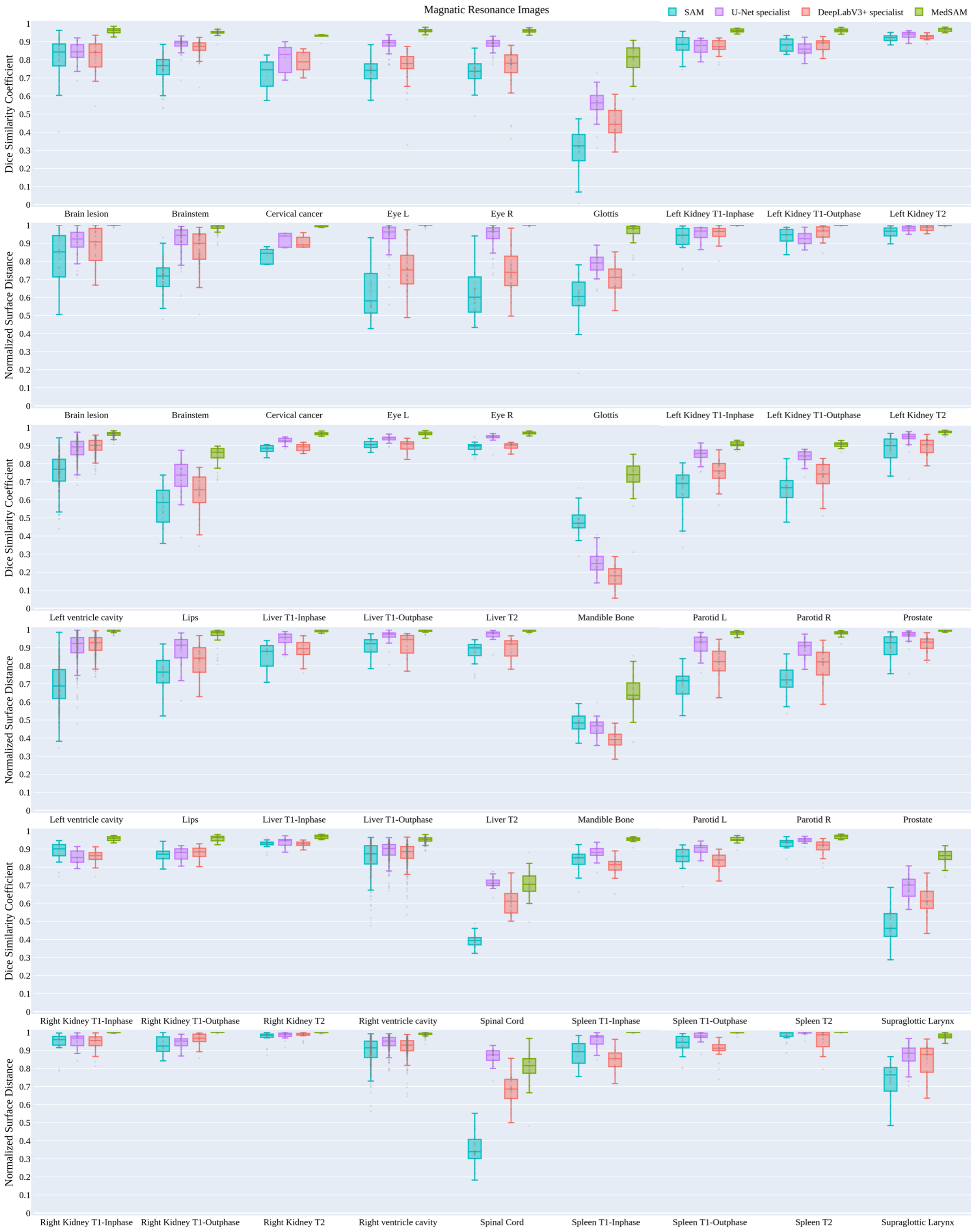

Fig. 11. Box plots of dice similarity coefficient and normalized surface distance scores for each MR segmentation task in external validation. The center line within the box represents the median value, with the bottom and top bounds of the box delineating the 25th and 75th percentiles, respectively. Whiskers are chosen to show the 1.5 of the interquartile range. Source data are provided as a Source Data file.

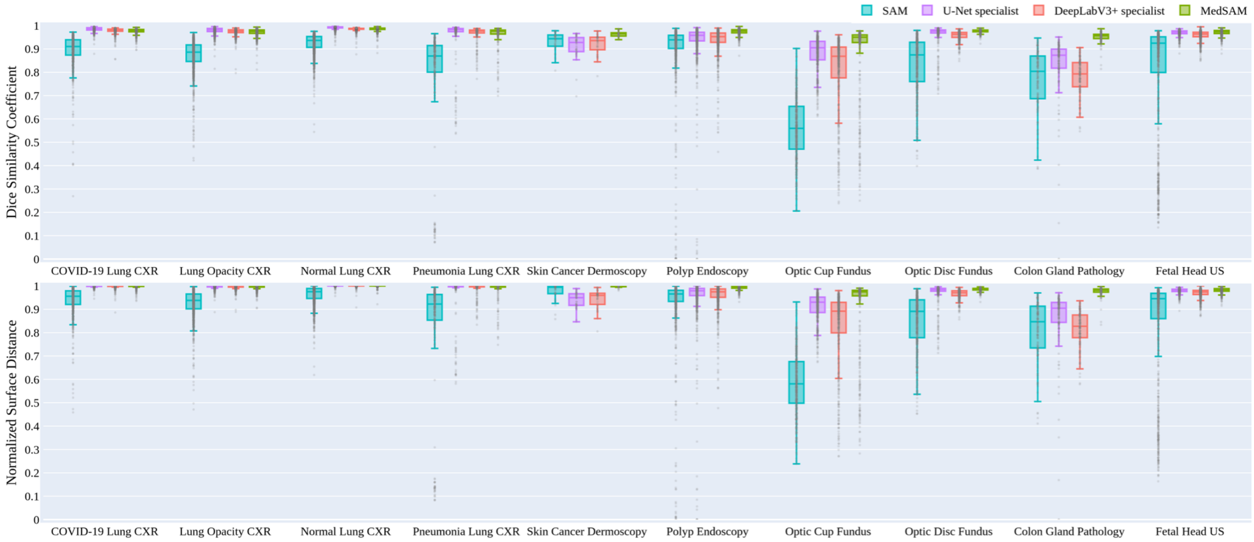

Fig. 12. Box plots of dice similarity coefficient and normalized surface distance scores for segmentation tasks of 2D modalities in external validation. The center line within the box represents the median value, with the bottom and top bounds of the box delineating the 25th and 75th percentiles, respectively. Whiskers are chosen to show the 1.5 of the interquartile range. Source data are provided as a Source Data file.

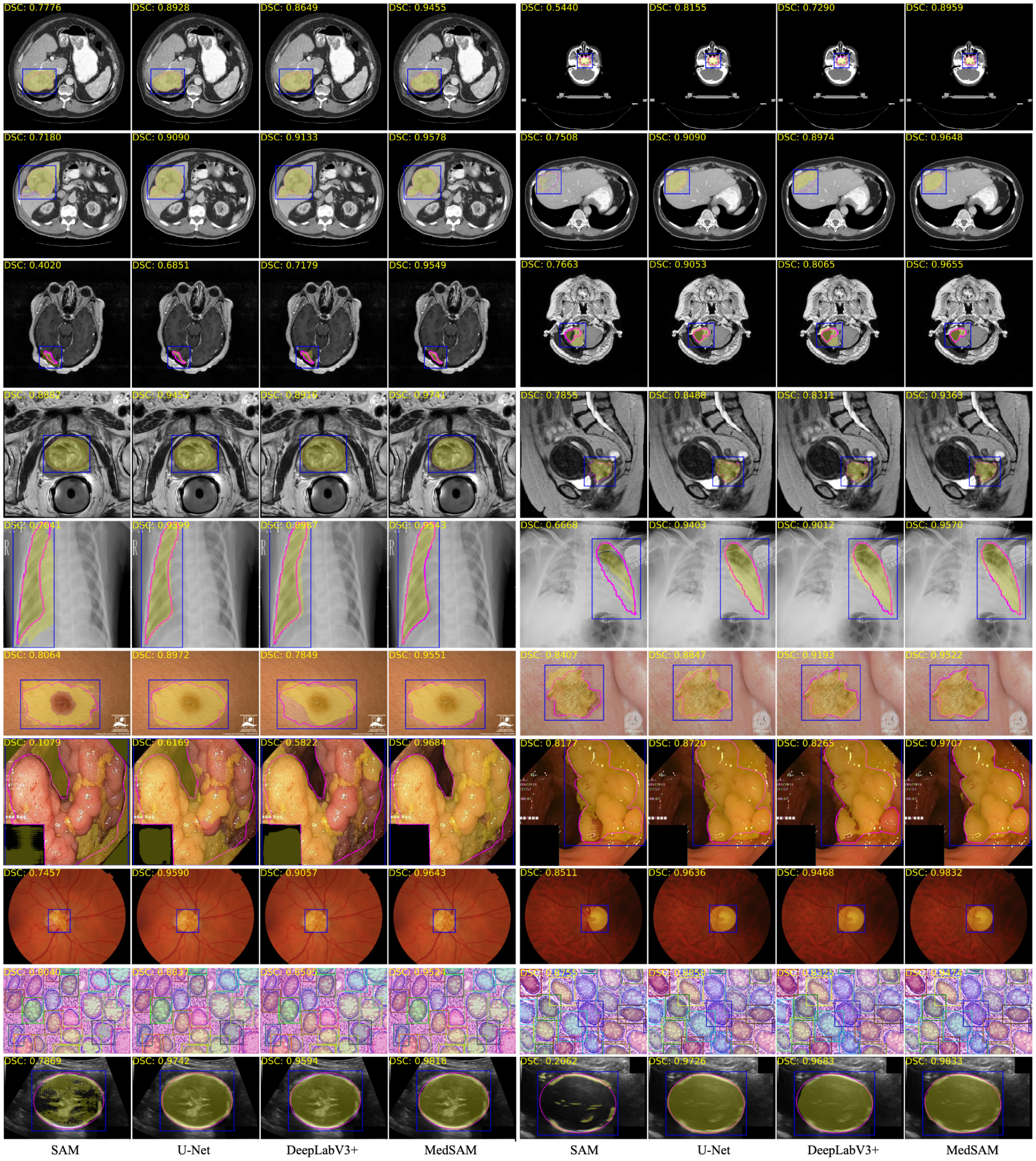

Fig. 13. Visualized segmentation examples in external validation. The visualized examples were randomly selected where MedSAM achieved a median DSC score for the corresponding task.

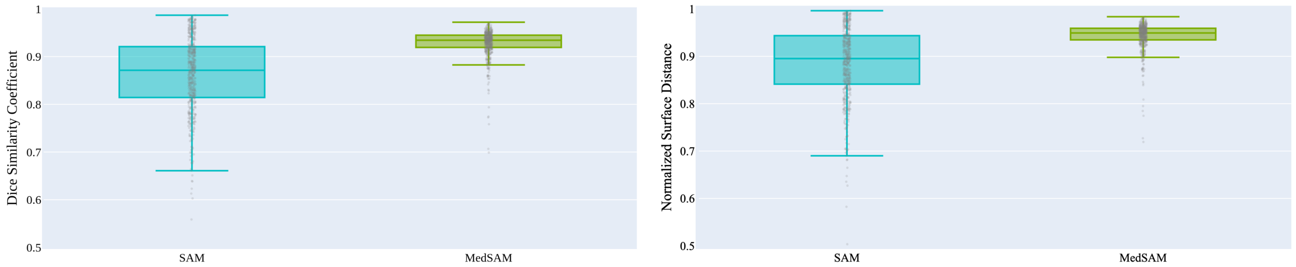

Fig. 14. Box plots of dice similarity coefficient and normalized surface distance scores for zero-shot experiments on multiple myeloma plasma cell segmentation, an unseen modality and task, demonstrate that MedSAM exhibits better generalization ability. The center line within the box represents the median value, with the bottom and top bounds of the box delineating the 25th and 75th percentiles, respectively; whiskers are chosen to show the 1.5 of the interquartile range. Source data are provided as a Source Data file.

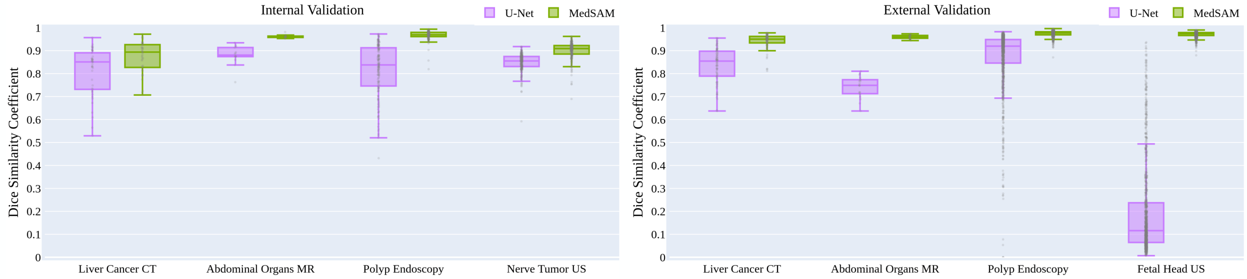

Fig. 15. Box-plot representation of comparison between task-specific U-Net models and MedSAM in terms of DSC scores. The center line within the box represents the median value, with the bottom and top bounds of the box delineating the 25th and 75th percentiles, respectively; whiskers are chosen to show the 1.5 of the interquartile range. While task-specific U-Net models often achieved great results on internal validation sets, their performance diminished significantly for external sets. In contrast, MedSAM maintained consistent performance across both internal and external validation sets. Source data are provided as a Source Data file.

TABLE 16
Data availability (CT and MR images).

Dataset	Modality	Download Link
AbdomenCT-1K [1], [2]	CT	https://github.com/JunMa11/AbdomenCT-1K
Adrenal-ACC-Ki67 [4]	CT	https://doi.org/10.7937/1FPG-VM46
AMOS-CT [6]	PET-CT	https://amos22.grand-challenge.org/
AutoPET [7]	CT	https://doi.org/10.7937/gkr0-xv29
COVID-19 Seg. Challenge [8], [9]	CT	https://covid-segmentation.grand-challenge.org/Data/
COVID-19-CT-Seg [10]	CT	https://github.com/JunMa11/COVID-19-CT-Seg-Benchmark
GLIS-RT [11]	CT	https://doi.org/10.7937/TCIA.T905-ZQ20
HCC-TACE-Seg [5], [12]	CT	https://doi.org/10.7937/TCIA.5FNA-0924
HECKTOR [13]	PET-CT	https://hecktor.grand-challenge.org/Overview/
INSTANCE [14]	CT	https://instance.grand-challenge.org/
KiPA [15], [16]	CT	https://kipa22.grand-challenge.org/
KiTS [17]	CT	https://kits-challenge.org/kits23/
LNQ2023 [18]	CT	https://lnq2023.grand-challenge.org/lnq2023/
Lymph Nodes [19], [20]	CT	https://doi.org/10.7937/K9/TCIA.2015.AQIIDCNM
MSD-Colon Tumor [21]	CT	http://medicaldecathlon.com/
MSD-Hepatic Tumor [21]	CT	http://medicaldecathlon.com/
MSD-Lung Tumor [21]	CT	http://medicaldecathlon.com/
MSD-Pancreas [21]	CT	http://medicaldecathlon.com/
MSD-Spleen [21]	CT	http://medicaldecathlon.com/
NSCLC Pleural Effusion [5], [22], [23]	CT	https://doi.org/10.7937/tcia.2020.6c7y-gq39
NSCLC Radiogenomics [24]	CT	https://doi.org/10.7937/K9/TCIA.2017.7hs46erv
ORG [25]	CT	https://www.nature.com/articles/s41597-020-00715-8
SegTHOR [26]	CT	https://competitions.codalab.org/competitions/21145
StructSeg [27]	CT	https://structseg2019.grand-challenge.org/Dataset/
TotalSegmentator [28]	CT	https://zenodo.org/record/6802614
WORD [29]	CT	https://github.com/HiLab-git/WORD
ACDC [30]	MR	https://humanheart-project.creatis.insa-lyon.fr/database/
AMOS-MR [6]	MR	https://amos22.grand-challenge.org/Dataset/
ATLAS R2.0 [31]	MR	https://atlas.grand-challenge.org/
Brain Tumor Dataset Figshare [32], [33]	MR	https://www.kaggle.com/datasets/ashkhagan/figshare-brain-tumor-dataset
Brain TR-GammakKnife [34]	MR	https://doi.org/10.7937/xb6d-py67
BraTS [35]–[39]	MR	http://braintumorsegmentation.org/
CC-Tumor Heterogeneity [40]	MR	https://doi.org/10.7937/ERZ5-QZ59
CHAOS [41]	MR	https://chaos.grand-challenge.org/
crossMoDA [42]	MR	https://crossmoda-challenge.ml/
FeTA [42]	MR	https://feta.grand-challenge.org/
HaN-Seg [43]	MR	https://zenodo.org/record/
ISLES [44]	MR	http://www.isles-challenge.org/
I2CVB [45]	MR	https://i2cvb.github.io/
Meningioma-SEG-CLASS [46]	MR	https://doi.org/10.7937/0TKV-1A36
MMs [47]	MR	https://www.ub.edu/mnms-2/
MSD-Heart [48]	MR	http://medicaldecathlon.com/
MSD-Prostate [48]	MR	http://medicaldecathlon.com/#tasks
NCI-ISBI [49]	MR	http://dx.doi.org/10.7937/K9/TCIA.2015.zF0vlOPv
PI-CAI [50]	MR	http://github.com/DIAGNijmegen/picai_labels
PPMI [51]	MR	https://www.ppmi-info.org/access-data-specimens/download-data
PROMISE [52]	MR	https://promise12.grand-challenge.org/Details/
Qin-Prostate-Repeatability [21], [53]	MR	http://doi.org/10.7937/K9/TCIA.2018.MR1CKGND
QUBIQ [54]	MR	https://qubiq21.grand-challenge.org/
Spine [55]	MR	https://www.cg.informatik.uni-siegen.de/en/spine-segmentation-and-analysis
WMH [56]	MR	https://wmh.isi.uu.nl/

TABLE 17
Data availability (Chest X-Ray (CXR), Dermoscopy, Endoscopy, Fundus, Mammography, Optical Coherence Tomography (OCT), Pathology, Ultrasound images).

Dataset	Modality	Download Link
Chest Xray Masks and Labels [57], [58]	CXR	https://www.kaggle.com/datasets/nikhilpandey360/chest-xray-masks-and-labels
Chest X-Ray (Pneumothorax) [59], [60]	CXR	https://www.kaggle.com/datasets/vbookshelf/pneumothorax-chest-xray-images-and-masks
COVID-19 Radiography [61], [62]	CXR	https://www.kaggle.com/datasets/tawsifurrahman/covid19-radiography-database
COVID-QU-Ex [61]–[65]	CXR	https://www.kaggle.com/datasets/anasmohammedtahir/covidqu
JSRT [66]	CXR	http://imgcom.jsrt.or.jp/minijsrtdb/
Lung [66], [68], [69]	CXR	https://www.kaggle.com/datasets/mohammadzunaed/lung-segmentation-datasets?select=ranzcr_clip
QaTa-COV19 [70]	CXR	https://www.kaggle.com/datasets/aysendegerli/qatacov19-dataset
ISIC [84]–[86]	Dermoscopy	https://challenge.isic-archive.com/data/
UWaterloo Skin Cancer [87]	Dermoscopy	https://uwaterloo.ca/vision-image-processing-lab/research-demos/skin-cancer-detection
BKAI-IGH NeoPolyp [88], [106]	Endoscopy	https://www.kaggle.com/competitions/bkai-igh-neopolyp/data
CholecSeg8k [89], [90]	Endoscopy	https://www.kaggle.com/datasets/newslab/cholecseg8k
Kvasir [91], [107]	Endoscopy	https://datasets.simula.no/kvasir/
m2caiSeg [92]	Endoscopy	https://www.kaggle.com/datasets/salmanmaq/m2caiseg
PolypGen [93]–[95]	Endoscopy	https://www.synapse.org/#!Synapse:syn45200214
RobTool [96]	Endoscopy	https://www.synapse.org/#!Synapse:syn22427422
sisvse [97]	Endoscopy	https://www.kaggle.com/datasets/yjh4374/sisvse-dataset
IDRiD [98]	Fundus	https://ieee-dataport.org/open-access/indian-diabetic-retinopathy-image-dataset-idrid
PAPILA [99]	Fundus	https://figshare.com/articles/dataset/PAPILA/14798004/1
REFUGE [100], [101]	Fundus	https://refuge.grand-challenge.org/
CDD-CESM [71], [72]	Mammography	https://doi.org/10.7937/29kw-ae92
Intraretinal Cystoid Fluid [73]	OCT	https://www.kaggle.com/datasets/zeeshanahmed13/intraretinal-cystoid-fluid
OCT Images (DME) [75]	OCT	https://www.kaggle.com/datasets/paultimothymooney/chiu-2015
GlaS@MICCAI2015 [102], [103]	Pathology	https://warwick.ac.uk/fac/cross_fac/tia/data/glascontest/download/
HuBMAP HPA	Pathology	https://www.kaggle.com/competitions/hubmap-organ-segmentation/
HuBMAP Hacking the Kidney	Pathology	https://www.kaggle.com/competitions/hubmap-kidney-segmentation/data?select=test
AbdomenUS [76]	Ultrasound	https://www.kaggle.com/datasets/ignaciorlando/ussimandsegm
Breast Cancer [77]	Ultrasound	https://www.kaggle.com/datasets/aryashah2k/breast-ultrasound-images-dataset
CAMUS [78]	Ultrasound	https://www.creatis.insa-lyon.fr/Challenge/camus/
CT2USforKidneySeg [79]	Ultrasound	https://www.kaggle.com/datasets/siatsyx/ct2usforkidneyseg
FH-PS-AOP [80]	Ultrasound	https://ps-fh-aop-2023.grand-challenge.org/
HC [81]	Ultrasound	https://hc18.grand-challenge.org/
TN-SCUI [82]	Ultrasound	https://tn-scui2020.grand-challenge.org/
Nerve	Ultrasound	https://www.kaggle.com/competitions/ultrasound-nerve-segmentation/data

References

[1] J. Ma, Y. Zhang, S. Gu, C. Zhu, C. Ge, Y. Zhang, X. An, C. Wang, Q. Wang, X. Liu, S. Cao, Q. Zhang, S. Liu, Y. Wang, Y. Li, J. He, and X. Yang, "Abdomenct-1k: Is abdominal organ segmentation a solved problem?" *IEEE Transactions on Pattern Analysis and Machine Intelligence*, 2021.

[2] J. Ma, Y. Zhang, S. Gu, X. An, Z. Wang, C. Ge, C. Wang, F. Zhang, Y. Wang, Y. Xu, S. Gou, F. Thaler, C. Payer, D. Štern, E. G. Henderson, D. M. McSweeney, A. Green, P. Jackson, L. McIntosh, Q.-C. Nguyen, A. Qayyum, P.-H. Conze, Z. Huang, Z. Zhou, D.-P. Fan, H. Xiong, G. Dong, Q. Zhu, J. He, and X. Yang, "Fast and low-gpu-memory abdomen ct organ segmentation: The flare challenge," *Medical Image Analysis*, vol. 82, p. 102616, 2022.

[3] A. W. Moawad, A. A. Ahmed, M. ElMohr, M. Eltaher, M. A. Habra, S. Fisher, N. Perrier, M. Zhang, D. Fuentes, and K. Elsayes, "Voxel-level segmentation of pathologically-proven adrenocortical carcinoma with ki-67 expression (adrenal-acc-ki67-seg) [data set]," https://doi.org/10.7937/1FPG-VM46, 2023.

[4] A. Ahmed, M. Elmohr, D. Fuentes, M. Habra, S. Fisher, N. Perrier, M. Zhang, and K. Elsayes, "Radiomic mapping model for prediction of ki-67 expression in adrenocortical carcinoma," *Clinical Radiology*, vol. 75, no. 6, pp. 479–e17, 2020.

[5] K. Clark, B. Vendt, K. Smith, J. Freymann, J. Kirby, P. Koppel, S. Moore, S. Phillips, D. Maffitt, M. Pringle *et al.*, "The cancer imaging archive (tcia): maintaining and operating a public information repository," *Journal of Digital Imaging*, vol. 26, no. 6, pp. 1045–1057, 2013.

[6] Y. Ji, H. Bai, J. Yang, C. Ge, Y. Zhu, R. Zhang, Z. Li, L. Zhang, W. Ma, X. Wan *et al.*, "Amos: A large-scale abdominal multi-organ benchmark for versatile medical image segmentation," *arXiv preprint arXiv:2206.08023*, 2022.

[7] S. Gatidis, T. Hepp, M. Früh, C. La Fougère, K. Nikolaou, C. Pfannenberg, B. Schölkopf, T. Küstner, C. Cyran, and D. Rubin, "A whole-body fdg-pet/ct dataset with manually annotated tumor lesions," *Scientific Data*, vol. 9, no. 1, p. 601, 2022.

[8] H. R. Roth, Z. Xu, C. Tor-Díez, R. S. Jacob, J. Zember, J. Molto, W. Li, S. Xu, B. Turkbey, E. Turkbey *et al.*, "Rapid artificial intelligence solutions in a pandemic—the covid-19-20 lung ct lesion segmentation challenge," *Medical Image Analysis*, vol. 82, p. 102605, 2022.

[9] P. An, S. Xu, S. Harmon, E. B. Turkbey, T. H. Sanford, A. Amalou, M. Kassin, N. Varble, M. Blain, V. Anderson *et al.*, "Ct images in covid-19 [data set]," *The Cancer Imaging Archive*, vol. 10, 2020.

[10] J. Ma, Y. Wang, X. An, C. Ge, Z. Yu, J. Chen, Q. Zhu, G. Dong, J. He, Z. He, T. Cao, Y. Zhu, Z. Nie, and X. Yang, "Towards data-efficient learning: A benchmark for covid-19 ct lung and infection segmentation," *Medical Physics*, vol. 48, no. 3, pp. 1197–1210, 2021.

[11] N. Shusharina, T. Bortfeld, C. Cardenas, B. De, K. Diao, S. Hernandez, Y. Liu, S. Maroongroge, J. Söderberg, and M. Soliman, "Cross-modality brain structures image segmentation for the radiotherapy target definition and plan optimization," in *Segmentation, Classification, and Registration of Multi-modality Medical Imaging Data: MICCAI 2020 Challenges, ABCs 2020, L2R 2020, TN-SCUI 2020, Held in Conjunction with MICCAI 2020, Lima, Peru, October 4–8, 2020, Proceedings 23*, 2021, pp. 3–15.

[12] A. Morshid, K. M. Elsayes, A. M. Khalaf, M. M. Elmohr, J. Yu, A. O. Kaseb, M. Hassan, A. Mahvash, Z. Wang, J. D. Hazle, and D. Fuentes, "A machine learning model to predict hepatocellular carcinoma response to transcatheter arterial chemoembolization," *Radiology: Artificial Intelligence*, vol. 1, no. 5, p. e180021, 2019.

[13] V. Oreiller, V. Andrearczyk, M. Jreige, S. Boughdad, H. Elhalawani, J. Castelli, M. Vallieres, S. Zhu, J. Xie, Y. Peng *et al.*, "Head and neck tumor segmentation in pet/ct: the hecktor challenge," *Medical Image Analysis*, vol. 77, p. 102336, 2022.

[14] X. Li, G. Luo, W. Wang, K. Wang, Y. Gao, and S. Li, "Hematoma expansion context guided intracranial hemorrhage segmentation and uncertainty estimation," *IEEE Journal of Biomedical and Health Informatics*, vol. 26, no. 3, pp. 1140–1151, 2021.

[15] Y. He, G. Yang, J. Yang, R. Ge, Y. Kong, X. Zhu, S. Zhang, P. Shao, H. Shu, J.-L. Dillenseger *et al.*, "Meta grayscale adaptive network for 3d integrated renal structures segmentation," *Medical Image Analysis*, vol. 71, p. 102055, 2021.

[16] Y. He, G. Yang, J. Yang, Y. Chen, Y. Kong, J. Wu, L. Tang, X. Zhu, J.-L. Dillenseger, P. Shao *et al.*, "Dense biased networks with deep priori anatomy and hard region adaptation: Semi-supervised learning for fine renal artery segmentation," *Medical Image Analysis*, vol. 63, p. 101722, 2020.

[17] N. Heller, F. Isensee, K. H. Maier-Hein, X. Hou, C. Xie, F. Li, Y. Nan, G. Mu, Z. Lin, M. Han *et al.*, "The state of the art in kidney and kidney tumor segmentation in contrast-enhanced ct imaging: Results of the kits19 challenge," *Medical Image Analysis*, vol. 67, p. 101821, 2021.

[18] R. Kikinis, S. Pieper, E. Ziegler, G. Harris, T. Idris, B. Somarouthu, H. Jacene, R. Khajavi, R. Dorent, and S. Pujol, "Mediastinal Lymph Node Quantification (LNQ): Segmentation of Heterogeneous CT Data," Apr. 2023. [Online]. Available: https://doi.org/10.5281/zenodo.7844666

[19] H. R. Roth, L. Lu, A. Seff, K. M. Cherry, J. Hoffman, S. Wang, J. Liu, E. Turkbey, and R. M. Summers, "A new 2.5 d representation for lymph node detection using random sets of deep convolutional neural network observations," in *Medical Image Computing and Computer-Assisted Intervention–MICCAI 2014: 17th International Conference, Boston, MA, USA, September 14-18, 2014, Proceedings, Part I 17*, 2014, pp. 520–527.

[20] H. Roth, L. Le, S. Ari, K. Cherry, J. Hoffman, S. Wang, and R. Summers, "A new 2.5 d representation for lymph node detection in ct," *The Cancer Imaging Archive*, 2018.

[21] M. Antonelli, A. Reinke, S. Bakas, K. Farahani, A. Kopp-Schneider, B. A. Landman, G. Litjens, B. Menze, O. Ronneberger, R. M. Summers *et al.*, "The medical segmentation decathlon," *Nature Communications*, vol. 13, no. 1, p. 4128, 2022.

[22] K. J. Kiser, S. Ahmed, S. Stieb, A. S. Mohamed, H. Elhalawani, P. Y. Park, N. S. Doyle, B. J. Wang, A. Barman, Z. Li *et al.*, "Plethora: Pleural effusion and thoracic cavity segmentations in diseased lungs for benchmarking chest ct processing pipelines," *Medical Physics*, vol. 47, no. 11, pp. 5941–5952, 2020.

[23] K. J. Kiser, S. Ahmed, S. M. Stieb, A. S. Mohamed, H. Elhalawani, P. Y. Park, N. S. Doyle, B. J. Wang, A. Barman, C. D. Fuller, and L. Giancardo, "Data from the thoracic volume and pleural effusion segmentations in diseased lungs for benchmarking chest ct processing pipelines (plethora)," Data set, 2020. [Online]. Available: https://doi.org/10.7937/tcia.2020.6c7y-gq39

[24] S. Bakr, O. Gevaert, S. Echegaray, K. Ayers, M. Zhou, M. Shafiq, H. Zheng, J. A. Benson, W. Zhang, A. N. Leung *et al.*, "A radiogenomic dataset of non-small cell lung cancer," *Scientific Data*, vol. 5, no. 1, pp. 1–9, 2018.

[25] B. Rister, D. Yi, K. Shivakumar, T. Nobashi, and D. L. Rubin, "Ct-org, a new dataset for multiple organ segmentation in computed tomography," *Scientific Data*, vol. 7, no. 1, p. 381, 2020.

[26] Z. Lambert, C. Petitjean, B. Dubray, and S. Kuan, "Segthor: Segmentation of thoracic organs at risk in ct images," in *2020 Tenth International Conference on Image Processing Theory, Tools and Applications (IPTA)*, 2020, pp. 1–6.

[27] "Structseg2019 grand challenge dataset," Website, retrieved from https://structseg2019.grand-challenge.org/.

[28] J. Wasserthal, M. Meyer, H.-C. Breit, J. Cyriac, S. Yang, and M. Segeroth, "Totalsegmentator: robust segmentation of 104 anatomical structures in ct images," *arXiv preprint arXiv:2208.05868*, 2022.

[29] X. Luo, W. Liao, J. Xiao, J. Chen, T. Song, X. Zhang, K. Li, D. N. Metaxas, G. Wang, and S. Zhang, "Word: A large scale dataset, benchmark and clinical applicable study for abdominal organ segmentation from ct image," *Medical Image Analysis*, vol. 82, p. 102642, 2022.

[30] O. Bernard, A. Lalande, C. Zotti, F. Cervenansky, X. Yang, P.-A. Heng, I. Cetin, K. Lekadir, O. Camara, M. A. G. Ballester *et al.*, "Deep learning techniques for automatic mri cardiac multi-structures segmentation and diagnosis: is the problem solved?" *IEEE Transactions on Medical Imaging*, vol. 37, no. 11, pp. 2514–2525, 2018.

[31] S.-L. Liew, B. P. Lo, M. R. Donnelly, A. Zavaliangos-Petropulu, J. N. Jeong, G. Barisano, A. Hutton, J. P. Simon, J. M. Juliano, A. Suri *et al.*, "A large, curated, open-source stroke neuroimaging dataset to improve lesion segmentation algorithms," *Scientific Data*, vol. 9, no. 1, p. 320, 2022.

[32] J. Cheng, W. Huang, S. Cao, R. Yang, W. Yang, Z. Yun, Z. Wang, and Q. Feng, "Enhanced performance of brain tumor classification via tumor region augmentation and partition," *PloS One*, vol. 10, no. 10, p. e0140381, 2015.

[33] J. Cheng, W. Yang, M. Huang, W. Huang, J. Jiang, Y. Zhou, R. Yang, J. Zhao, Y. Feng, Q. Feng *et al.*, "Retrieval of brain tumors by adaptive spatial pooling and fisher vector representation," *PloS One*, vol. 11, no. 6, p. e0157112, 2016.

[34] Y. Wang, W. Duggar, D. Caballero, T. Vengaloor Thomas, N. Adari, E. Mundra, and H. Wang, "Brain tumor recurrence prediction after gamma knife radiotherapy from mri and related dicom-rt: An open annotated dataset and baseline algorithm (brain-tr-gammaknife) [dataset]," The Cancer Imaging Archive, 2023.

[35] B. H. Menze, A. Jakab, S. Bauer, J. Kalpathy-Cramer, K. Farahani, J. Kirby, Y. Burren, N. Porz, J. Slotboom, R. Wiest *et al.*, "The multimodal brain tumor image segmentation benchmark (brats)," *IEEE Transactions on Medical Imaging*, vol. 34, no. 10, pp. 1993–2024, 2014.

[36] S. Bakas, M. Reyes, A. Jakab, S. Bauer, M. Rempfler, A. Crimi *et al.*, "Identifying the best machine learning algorithms for brain tumor segmentation, progression assessment, and overall survival prediction in the brats challenge," *arXiv preprint arXiv:1811.02629*, 2018.

[37] S. Bakas, H. Akbari, A. Sotiras, M. Bilello, M. Rozycki, J. S. Kirby, J. B. Freymann, K. Farahani, and C. Davatzikos, "Advancing the cancer genome atlas glioma mri collections with expert segmentation labels and radiomic features," *Scientific data*, vol. 4, p. 170117, 2017.

[38] B. Spyridon, A. Hamed, S. Aristeidis, B. Michel, R. Martin, K. Justin, F. John, F. Keyvan, and D. Christos, "Segmentation labels and radiomic features for the pre-operative scans of the tcga-gbm collection," *The Cancer Imaging Archive*, 2017.

[39] ——, "Segmentation labels and radiomic features for the pre-operative scans of the tcga-lgg collection," *The Cancer Imaging Archive*, 2017.

[40] S. R. Bowen, W. T. Yuh, D. S. Hippe, W. Wu, S. C. Partridge, S. Elias, G. Jia, Z. Huang, G. A. Sandison, D. Nelson *et al.*, "Tumor radiomic heterogeneity: multiparametric functional imaging to characterize variability and predict response following cervical cancer radiation therapy," *Journal of Magnetic Resonance Imaging*, vol. 47, no. 5, pp. 1388–1396, 2018.

[41] A. E. Kavur, N. S. Gezer, M. Barış, S. Aslan, P.-H. Conze, V. Groza, D. D. Pham, S. Chatterjee, P. Ernst, S. Özkan, B. Baydar, D. Lachinov, S. Han, J. Pauli, F. Isensee, M. Perkonigg, R. Sathish, R. Rajan, D. Sheet, G. Dovletov, O. Speck, A. Nürnberger, K. H. Maier-Hein, G. Bozdağı Akar, G. Ünal, O. Dicle, and M. A. Selver, "CHAOS Challenge - combined (CT-MR) healthy abdominal organ segmentation," *Medical Image Analysis*, vol. 69, p. 101950, 2021. [Online]. Available: http://www.sciencedirect.com/science/article/pii/S1361841520303145

[42] R. Dorent, A. Kujawa, M. Ivory, S. Bakas, N. Rieke, S. Joutard, B. Glocker, J. Cardoso, M. Modat, K. Batmanghelich *et al.*, "Crossmoda 2021 challenge: Benchmark of cross-modality domain adaptation techniques for vestibular schwannoma and cochlea segmentation," *Medical Image Analysis*, vol. 83, p. 102628, 2023.

[43] G. Podobnik, P. Strojan, P. Peterlin, B. Ibragimov, and T. Vrtovec, "Han-seg: The head and neck organ-at-risk ct & mr segmentation dataset," *Medical Physics*, 2023.

[44] M. R. Hernandez Petzsche, E. de la Rosa, U. Hanning, R. Wiest, W. Valenzuela, M. Reyes, M. Meyer, S.-L. Liew, F. Kofler, I. Ezhov *et al.*, "Isles 2022: A multi-center magnetic resonance imaging stroke lesion segmentation dataset," *Scientific Data*, vol. 9, no. 1, p. 762, 2022.

[45] R. Trigui, J. Mitéran, P. M. Walker, L. Sellami, and A. B. Hamida, "Automatic classification and localization of prostate cancer using multi-parametric mri/mrs," *Biomedical Signal Processing and Control*, vol. 31, pp. 189–198, 2017.

[46] A. Vassantachart, Y. Cao, M. Gribble, S. Guzman, J. C. Ye, K. Hurth, A. Mathew, G. Zada, Z. Fan, E. L. Chang *et al.*, "Automatic differentiation of grade i and ii meningiomas on magnetic resonance image using an asymmetric convolutional neural network," *Scientific Reports*, vol. 12, no. 1, p. 3806, 2022.

[47] V. M. Campello, P. Gkontra, C. Izquierdo, C. Martin-Isla, A. Sojoudi, P. M. Full, K. Maier-Hein, Y. Zhang, Z. He, J. Ma *et al.*, "Multi-centre, multi-vendor and multi-disease cardiac segmentation: the m&ms challenge," *IEEE Transactions on Medical Imaging*, vol. 40, no. 12, pp. 3543–3554, 2021.

[48] A. L. Simpson, M. Antonelli, S. Bakas, M. Bilello, K. Farahani, B. Van Ginneken, A. Kopp-Schneider, B. A. Landman, G. Litjens, B. Menze *et al.*, "A large annotated medical image dataset for the development and evaluation of segmentation algorithms," *arXiv preprint arXiv:1902.09063*, 2019.

[49] N. Bloch, A. Madabhushi, H. Huisman, J. Freymann, J. Kirby, M. Grauer, A. Enquobahrie, C. Jaffe, L. Clarke, and K. Farahani, "Nci-isbi 2013 challenge: Automated segmentation of prostate structures," *The Cancer Imaging Archive*, 2015.

[50] A. Saha, M. Hosseinzadeh, and H. Huisman, "End-to-end prostate cancer detection in bpmri via 3d cnns: effects of attention mechanisms, clinical priori and decoupled false positive reduction," *Medical Image Analysis*, vol. 73, p. 102155, 2021.

[51] K. Marek, D. Jennings, S. Lasch, A. Siderowf, C. Tanner, T. Simuni, C. Coffey, K. Kieburtz, E. Flagg, S. Chowdhury *et al.*, "The parkinson progression marker initiative (ppmi)," *Progress in Neurobiology*, vol. 95, no. 4, pp. 629–635, 2011.

[52] G. Litjens, R. Toth, W. Van De Ven, C. Hoeks, S. Kerkstra, B. van Ginneken, G. Vincent, G. Guillard, N. Birbeck, J. Zhang *et al.*, "Evaluation of prostate segmentation algorithms for mri: the promise12 challenge," *Medical Image Analysis*, vol. 18, no. 2, pp. 359–373, 2014.

[53] A. Fedorov, M. Schwier, D. Clunie, C. Herz, S. Pieper, R. Kikinis, C. Tempany, and F. Fennessy, "An annotated test-retest collection of prostate multiparametric mri," *Scientific Data*, vol. 5, no. 1, pp. 1–13, 2018.

[54] A. S. Becker, K. Chaitanya, K. Schawkat, U. J. Muehlematter, A. M. Hötker, E. Konukoglu, and O. F. Donati, "Variability of manual segmentation of the prostate in axial t2-weighted mri: A multi-reader study," *European Journal of Radiology*, vol. 121, p. 108716, 2019.

[55] D. Zukić, A. Vlasák, J. Egger, D. Hořínek, C. Nimsky, and A. Kolb, "Robust detection and segmentation for diagnosis of vertebral diseases using routine mr images," in *Computer Graphics Forum*, vol. 33, no. 6, 2014, pp. 190–204.

[56] H. J. Kuijf, J. M. Biesbroek, J. De Bresser, R. Heinen, S. Andermatt, M. Bento, M. Berseth, M. Belyaev, M. J. Cardoso, A. Casamitjana *et al.*, "Standardized assessment of automatic segmentation of white matter hyperintensities and results of the wmh segmentation challenge," *IEEE Transactions on Medical Imaging*, vol. 38, no. 11, pp. 2556–2568, 2019.

[57] S. Jaeger, A. Karargyris, S. Candemir, L. Folio, J. Siegelman, F. Callaghan, Z. Xue, K. Palaniappan, R. K. Singh, S. Antani *et al.*, "Automatic tuberculosis screening using chest radiographs," *IEEE Transactions on Medical Imaging*, vol. 33, no. 2, pp. 233–245, 2013.

[58] S. Candemir, S. Jaeger, K. Palaniappan, J. P. Musco, R. K. Singh, Z. Xue, A. Karargyris, S. Antani, G. Thoma, and C. J. McDonald, "Lung segmentation in chest radiographs using anatomical atlases with nonrigid registration," *IEEE Transactions on Medical Imaging*, vol. 33, no. 2, pp. 577–590, 2013.

[59] vbookshelf, "Chest x-ray images with pneumothorax masks," https://www.kaggle.com/datasets/vbookshelf/pneumothorax-chest-xray-images-and-masks, 2020.

[60] S. for Imaging Informatics in Medicine (SIIM), "Siim-acr pneumothorax segmentation," https://www.kaggle.com/competitions/siim-acr-pneumothorax-segmentation/overview, 2019.

[61] T. Rahman, A. Khandakar, Y. Qiblawey, A. Tahir, S. Kiranyaz, S. B. A. Kashem, M. T. Islam, S. Al Maadeed, S. M. Zughaier, M. S. Khan *et al.*, "Exploring the effect of image enhancement techniques on covid-19 detection using chest x-ray images," *Computers in Biology and Medicine*, vol. 132, p. 104319, 2021.

[62] M. E. Chowdhury, T. Rahman, A. Khandakar, R. Mazhar, M. A. Kadir, Z. B. Mahbub, K. R. Islam, M. S. Khan, A. Iqbal, N. Al Emadi *et al.*, "Can ai help in screening viral and covid-19 pneumonia?" *Ieee Access*, vol. 8, pp. 132 665–132 676, 2020.

[63] A. M. Tahir, M. E. Chowdhury, A. Khandakar, T. Rahman, Y. Qiblawey, U. Khurshid, S. Kiranyaz, N. Ibtehaz, M. S. Rahman, S. Al-Maadeed *et al.*, "Covid-19 infection localization and severity grading from chest x-ray images," *Computers in Biology and Medicine*, vol. 139, p. 105002, 2021.

[64] A. Degerli, M. Ahishali, M. Yamac, S. Kiranyaz, M. E. Chowdhury, K. Hameed, T. Hamid, R. Mazhar, and M. Gabbouj, "Covid-19 infection map generation and detection from chest x-ray images," *Health Information Science and Systems*, vol. 9, no. 1, p. 15, 2021.

[65] A. M. Tahir, M. E. H. Chowdhury, Y. Qiblawey, A. Khandakar, T. Rahman, S. Kiranyaz, U. Khurshid, N. Ibtehaz, S. Mahmud, and M. Ezeddin, "Covid-qu-ex dataset," 2022.

[66] J. Shiraishi, S. Katsuragawa, J. Ikezoe, T. Matsumoto, T. Kobayashi, K.-i. Komatsu, M. Matsui, H. Fujita, Y. Kodera, and K. Doi, "Development of a digital image database for chest radiographs with and without a lung nodule: receiver operating characteristic analysis of radiologists' detection of pulmonary nodules," *American Journal of Roentgenology*, vol. 174, no. 1, pp. 71–74, 2000.

[67] ——, "Development of a digital image database for chest radiographs with and without a lung nodule," *American Journal of Roentgenology*, vol. 174, no. 1, pp. 71–74, 2000.

[68] B. van Ginneken, M. B. Stegmann, and M. Loog, "Segmentation of anatomical structures in chest radiographs using supervised methods: a comparative study on a public database," *Medical Image Analysis*, vol. 10, no. 1, pp. 19–40, 2006.

[69] D. Barušauskas. Ranzcr Clip lung contours. [Online]. Available: https://www.kaggle.com/datasets/raddar/ranzcr-clip-lung-contours

[70] A. Degerli, S. Kiranyaz, M. E. Chowdhury, and M. Gabbouj, "Osegnet: Operational segmentation network for covid-19 detection using chest x-ray images," in *2022 IEEE International Conference on Image Processing (ICIP)*, 2022, pp. 2306–2310.

[71] R. Khaled, M. Helal, O. Alfarghaly, O. Mokhtar, A. Elkorany, H. El Kassas, and A. Fahmy, "Categorized contrast enhanced mammography dataset for diagnostic and artificial intelligence research," *Scientific Data*, vol. 9, no. 1, p. 122, 2022.

[72] ——, "Categorized digital database for low energy and subtracted contrast enhanced spectral mammography images [dataset]," 2021.

[73] Z. Ahmed, S. Q. Panhwar, A. Baqai, F. A. Umrani, M. Ahmed, and A. Khan, "Deep learning based automated detection of intraretinal cystoid fluid," *International Journal of Imaging Systems and Technology*, vol. 32, no. 3, pp. 902–917, 2022.

[74] Z. Ahmed, M. Ahmed, A. Baqai, and F. A. Umrani, "Intraretinal cystoid fluid," 2022.

[75] S. J. Chiu, M. J. Allingham, P. S. Mettu, S. W. Cousins, J. A. Izatt, and S. Farsiu, "Kernel regression based segmentation of optical coherence tomography images with diabetic macular edema," *Biomedical Optics Express*, vol. 6, no. 4, pp. 1172–1194, 2015.

[76] S. Vitale, J. I. Orlando, E. Iarussi, and I. Larrabide, "Improving realism in patient-specific abdominal ultrasound simulation using cyclegans," *International Journal of Computer Assisted Radiology and Surgery*, vol. 15, no. 2, pp. 183–192, 2020.

[77] W. Al-Dhabyani, M. Gomaa, H. Khaled, and A. Fahmy, "Dataset of breast ultrasound images," *Data in Brief*, vol. 28, p. 104863, 2020.

[78] S. Leclerc, E. Smistad, J. Pedrosa, A. Østvik, F. Cervenansky, F. Espinosa, T. Espeland, E. A. R. Berg, P.-M. Jodoin, T. Grenier *et al.*, "Deep learning for segmentation using an open large-scale dataset in 2d echocardiography," *IEEE Transactions on Medical Imaging*, vol. 38, no. 9, pp. 2198–2210, 2019.

[79] Y. Song, J. Zheng, L. Lei, Z. Ni, B. Zhao, and Y. Hu, "Ct2us: Cross-modal transfer learning for kidney segmentation in ultrasound images with synthesized data," *Ultrasonics*, vol. 122, p. 106706, 2022.

[80] Y. Lu, M. Zhou, D. Zhi, M. Zhou, X. Jiang, R. Qiu, Z. Ou, H. Wang, D. Qiu, M. Zhong *et al.*, "The jnu-ifm dataset for segmenting pubic symphysis-fetal head," *Data in Brief*, vol. 41, p. 107904, 2022.

[81] T. L. van den Heuvel, D. de Bruijn, C. L. de Korte, and B. v. Ginneken, "Automated measurement of fetal head circumference using 2d ultrasound images," *PloS One*, vol. 13, no. 8, p. e0200412, 2018.

[82] J. Zhou, X. Jia, Y. Dong, D. Ni, A. Noble, R. Huang, T. Tan, X. Tao, R. Li, and M. The Van, "Thyroid nodule segmentation and classification," https://tn-scui2020.grand-challenge.org/Home/, 2020.

[83] Kaggle, "Ultrasound nerve segmentation," 2016.

[84] P. Tschandl, C. Rosendahl, and H. Kittler, "The ham10000 dataset, a large collection of multi-source dermatoscopic images of common pigmented skin lesions," *Scientific Data*, vol. 5, no. 1, pp. 1–9, 2018.

[85] N. C. Codella, D. Gutman, M. E. Celebi, B. Helba, M. A. Marchetti, S. W. Dusza, A. Kalloo, K. Liopyris, N. Mishra, H. Kittler *et al.*, "Skin lesion analysis toward melanoma detection: A challenge at the 2017 international symposium on biomedical imaging (isbi), hosted by the international skin imaging collaboration (isic)," in *2018 IEEE 15th International Symposium on Biomedical Imaging (ISBI 2018)*, 2018, pp. 168–172.

[86] N. Codella, V. Rotemberg, P. Tschandl, M. E. Celebi, S. Dusza, D. Gutman, B. Helba, A. Kalloo, K. Liopyris, M. Marchetti *et al.*, "Skin lesion analysis toward melanoma detection 2018: A challenge hosted by the international skin imaging collaboration (isic)," *arXiv preprint arXiv:1902.03368*, 2019.

[87] University of Waterloo, "Skin Cancer Detection," https://uwaterloo.ca/vision-image-processing-lab/research-demos/skin-cancer-detection.

[88] P. Ngoc Lan, N. S. An, D. V. Hang, D. V. Long, T. Q. Trung, N. T. Thuy, and D. V. Sang, "Neounet: Towards accurate colon polyp segmentation and neoplasm detection," in *Advances in Visual Computing: 16th International Symposium, ISVC 2021, Virtual Event, October 4-6, 2021, Proceedings, Part II*, 2021, pp. 15–28.

[89] W.-Y. Hong, C.-L. Kao, Y.-H. Kuo, J.-R. Wang, W.-L. Chang, and C.-S. Shih, "Cholecseg8k: a semantic segmentation dataset for laparoscopic cholecystectomy based on cholec80," *arXiv preprint arXiv:2012.12453*, 2020.

[90] A. P. Twinanda, S. Shehata, D. Mutter, J. Marescaux, M. De Mathelin, and N. Padoy, "Endonet: a deep architecture for recognition tasks on laparoscopic videos," *IEEE Transactions on Medical Imaging*, vol. 36, no. 1, pp. 86–97, 2016.

[91] D. Jha, P. H. Smedsrud, M. A. Riegler, P. Halvorsen, T. de Lange, D. Johansen, and H. D. Johansen, "Kvasir-seg: A segmented polyp dataset," in *MultiMedia Modeling: 26th International Conference, MMM 2020, Daejeon, South Korea, January 5–8, 2020, Proceedings, Part II 26*, 2020, pp. 451–462.

[92] S. Maqbool, A. Riaz, H. Sajid, and O. Hasan, "m2caiseg: Semantic segmentation of laparoscopic images using convolutional neural networks," *arXiv preprint arXiv:2008.10134*, 2020.

[93] S. Ali, D. Jha, N. Ghatwary, S. Realdon, R. Cannizzaro, O. E. Salem, D. Lamarque, C. Daul, M. A. Riegler, K. V. Anonsen *et al.*, "Polypgen: A multi-center polyp detection and segmentation dataset for generalisability assessment," *arXiv preprint arXiv:2106.04463*, 2021.

[94] S. Ali, N. Ghatwary, D. Jha, E. Isik-Polat, G. Polat, C. Yang, W. Li, A. Galdran, M.-Á. G. Ballester, V. Thambawita *et al.*, "Assessing generalisability of deep learning-based polyp detection and segmentation methods through a computer vision challenge," *arXiv preprint arXiv:2202.12031*, 2022.

[95] S. Ali, M. Dmitrieva, N. Ghatwary, S. Bano, G. Polat, A. Temizel, A. Krenzer, A. Hekalo, Y. B. Guo, B. Matuszewski *et al.*, "Deep learning for detection and segmentation of artefact and disease instances in gastrointestinal endoscopy," *Medical Image Analysis*, vol. 70, p. 102002, 2021.

[96] L. C. Garcia-Peraza-Herrera, L. Fidon, C. D'Ettorre, D. Stoyanov, T. Vercauteren, and S. Ourselin, "Image compositing for segmentation of surgical tools without manual annotations," *IEEE Transactions on Medical Imaging*, vol. 40, no. 5, pp. 1450–1460, 2021.

[97] J. Yoon, S. Hong, S. Hong, J. Lee, S. Shin, B. Park, N. Sung, H. Yu, S. Kim, S. Park *et al.*, "Surgical scene segmentation using semantic image synthesis with a virtual surgery environment," in *Medical Image Computing and Computer Assisted Intervention–MICCAI 2022: 25th International Conference, Singapore, September 18–22, 2022, Proceedings, Part VII*, 2022, pp. 551–561.

[98] P. Porwal, S. Pachade, R. Kamble, M. Kokare, G. Deshmukh, V. Sahasrabuddhe, and F. Meriaudeau, "Indian diabetic retinopathy image dataset (idrid): a database for diabetic retinopathy screening research," *Data*, vol. 3, no. 3, p. 25, 2018.

[99] O. Kovalyk, J. Morales-Sánchez, R. Verdú-Monedero, I. Sellés-Navarro, A. Palazón-Cabanes, and J.-L. Sancho-Gómez, "Papila: Dataset with fundus images and clinical data of both eyes of the same patient for glaucoma assessment," *Scientific Data*, vol. 9, no. 1, p. 291, 2022.

[100] F. Li, D. Song, H. Chen, J. Xiong, X. Li, H. Zhong, G. Tang, S. Fan, D. S. Lam, W. Pan *et al.*, "Development and clinical deployment of a smartphone-based visual field deep learning system for glaucoma detection," *NPJ Digital Medicine*, vol. 3, no. 1, p. 123, 2020.

[101] J. I. Orlando, H. Fu, J. B. Breda, K. Van Keer, D. R. Bathula, A. Diaz-Pinto, R. Fang, P.-A. Heng, J. Kim, J. Lee *et al.*, "Refuge challenge: A unified framework for evaluating automated methods for glaucoma assessment from fundus photographs," *Medical Image Analysis*, vol. 59, p. 101570, 2020.

[102] K. Sirinukunwattana, D. R. Snead, and N. M. Rajpoot, "A stochastic polygons model for glandular structures in colon histology images," *IEEE Transactions on Medical Imaging*, vol. 34, no. 11, pp. 2366–2378, 2015.

[103] K. Sirinukunwattana, J. P. Pluim, H. Chen, X. Qi, P.-A. Heng, Y. B. Guo, L. Y. Wang, B. J. Matuszewski, E. Bruni, U. Sanchez *et al.*, "Gland segmentation in colon histology images: The glas challenge contest," *Medical Image Analysis*, vol. 35, pp. 489–502, 2017.

[104] HuBMAP + HPA, "HuBMAP + HPA - Hacking the Human Body," https://www.kaggle.com/competitions/hubmap-organ-segmentation, 2022.

[105] InnovationDigi, "HuBMAP - Hacking the Kidney: Identify glomeruli in human kidney tissue images," https://www.kaggle.com/competitions/hubmap-kidney-segmentation/data?select=test, 2021.

[106] N. S. An, P. N. Lan, D. V. Hang, D. V. Long, T. Q. Trung, N. T. Thuy, and D. V. Sang, "Blazeneo: Blazing fast polyp segmentation and neoplasm detection," *IEEE Access*, vol. 10, pp. 43 669–43 684, 2022.

[107] K. Pogorelov, K. R. Randel, C. Griwodz, S. L. Eskeland, T. de Lange, D. Johansen, C. Spampinato, D.-T. Dang-Nguyen, M. Lux, P. T. Schmidt *et al.*, "Kvasir: A multi-class image dataset for computer aided gastrointestinal disease detection," in *Proceedings of the 8th ACM on Multimedia Systems Conference*, 2017, pp. 164–169.
