# Supplementary material for: Segment anything in medical images
Source: Nat Commun. 2024 Jan 22;15:654. doi: 10.1038/s41467-024-44824-z (PMC10803759; doi:10.1038/s41467-024-44824-z)
Supplement: Supplementary file 2 — Reporting Summary [file 41467_2024_44824_MOESM2_ESM.pdf]

nature portfolio

Corresponding author(s): Bo Wang

Last updated by author(s): Nov 28, 2023

Reporting Summary

Nature Portfolio wishes to improve the reproducibility of the work that we publish. This form provides structure for consistency and transparency in reporting. For further information on Nature Portfolio policies, see our Editorial Policies and the Editorial Policy Checklist.

Statistics

For all statistical analyses, confirm that the following items are present in the figure legend, table legend, main text, or Methods section.

n/a	Confirmed	
☐	☒	The exact sample size (n) for each experimental group/condition, given as a discrete number and unit of measurement
☐	☒	A statement on whether measurements were taken from distinct samples or whether the same sample was measured repeatedly
☐	☒	The statistical test(s) used AND whether they are one- or two-sided<br>*Only common tests should be described solely by name; describe more complex techniques in the Methods section.*
☒	☐	A description of all covariates tested
☒	☐	A description of any assumptions or corrections, such as tests of normality and adjustment for multiple comparisons
☐	☒	A full description of the statistical parameters including central tendency (e.g. means) or other basic estimates (e.g. regression coefficient) AND variation (e.g. standard deviation) or associated estimates of uncertainty (e.g. confidence intervals)
☐	☒	For null hypothesis testing, the test statistic (e.g. F, t, r) with confidence intervals, effect sizes, degrees of freedom and P value noted<br>*Give P values as exact values whenever suitable.*
☒	☐	For Bayesian analysis, information on the choice of priors and Markov chain Monte Carlo settings
☒	☐	For hierarchical and complex designs, identification of the appropriate level for tests and full reporting of outcomes
☒	☐	Estimates of effect sizes (e.g. Cohen's d, Pearson's r), indicating how they were calculated

Our web collection on statistics for biologists contains articles on many of the points above.

Software and code

Policy information about availability of computer code

Data collection	Image data for training and validation were collected with granted permission from the public domains, including the Cancer Imaging Archive, Kaggle, Grand-Challenge, Scientific Data, CodaLab, and segmentation challenges in the Medical Image Computing and Computer Assisted Intervention Society (MICCAI). An exhaustive list of datasets used in our study can be found and accessed in Supplementary Table 16-17.
Data analysis	All code was implemented in Python (3.10) using Pytorch (2.0) as the base deep learning framework. We also used several python packages for data analysis and results visualization, including SimpleITK (2.2.1), nibabel (5.1.0), torchvision (0.15.2), numpy (1.24.3), scikit-image (0.20.0), scipy (1.10.1), and pandas (2.0.2), matplotlib (3.7.1), opencv-python (4.8.0), ChallengeR (1.0.5), and plotly (5.15.0). Biorender was used to create Fig. 1

For manuscripts utilizing custom algorithms or software that are central to the research but not yet described in published literature, software must be made available to editors and reviewers. We strongly encourage code deposition in a community repository (e.g. GitHub). See the Nature Portfolio guidelines for submitting code & software for further information.

April 2023

Data

Policy information about availability of data

All manuscripts must include a data availability statement. This statement should provide the following information, where applicable:

- Accession codes, unique identifiers, or web links for publicly available datasets
- A description of any restrictions on data availability
- For clinical datasets or third party data, please ensure that the statement adheres to our policy

The datasets used in this study are available in the public domain and can be accessed via the links provided in Supplementary Table 16-17. Source data are provided with this paper in the Source Data file.

Research involving human participants, their data, or biological material

Policy information about studies with human participants or human data. See also policy information about sex, gender (identity/presentation), and sexual orientation and race, ethnicity and racism.

Reporting on sex and gender	All reported findings apply to patients of any sex or gender.
Reporting on race, ethnicity, or other socially relevant groupings	This study involves no recruitment of human participants
Population characteristics	This study involves no recruitment of human participants
Recruitment	This study involves no recruitment of human participants
Ethics oversight	This study involves no recruitment of human participants

Note that full information on the approval of the study protocol must also be provided in the manuscript.

Field-specific reporting

Please select the one below that is the best fit for your research. If you are not sure, read the appropriate sections before making your selection.

☒ Life sciences ☐ Behavioural & social sciences ☐ Ecological, evolutionary & environmental sciences

For a reference copy of the document with all sections, see nature.com/documents/nr-reporting-summary-flat.pdf

Life sciences study design

All studies must disclose on these points even when the disclosure is negative.

Sample size	No sample size calculation was made. For training and internal validation, we performed a 8:1:1 split on the internal datasets for training, tuning and validating. For external validation, we applied exclusion criteria for quality control; details regarding the criteria can be found under the Method section. An exhaustive list of sample sizes used per dataset can be found in Supplementary Table 1-4. An exhaustive list of sample sizes used per modality can be found in Supplementary Table 5.
Data exclusions	Several exclusive criteria are applied to improve the dataset quality and consistency, including incomplete images and segmentation targets with branching structures, inaccurate annotations, and tiny volumes.
Replication	We performed independent external validation on new datasets; code and trained models have been made publicly available for reproducing the reported results.
Randomization	In internal validation, the dataset was randomly divided into training, tuning and validation sets, with proportions of 80%, 10% and 10% respectively.
Blinding	In this computational study, experimental groups per se did not exist and thus blinding was irrelevant to this study. However, in keeping with good practice, allocation of training, tuning and validation sets was performed randomly by computer algorithm and blinded to the experimenters. In the internal dataset, a video-based split was adopted for images sourced from endoscopy videos, and a case-based split was adopted for 3D image modalities such as CT and MR, as opposed to image-wise, for the training, tuning, and validation sets to avert data leakage. All experiments performed in silico by AI did not require blinding.

Reporting for specific materials, systems and methods

We require information from authors about some types of materials, experimental systems and methods used in many studies. Here, indicate whether each material, system or method listed is relevant to your study. If you are not sure if a list item applies to your research, read the appropriate section before selecting a response.

Materials & experimental systems

n/a	Involved in the study
☒	☐ Antibodies
☒	☐ Eukaryotic cell lines
☒	☐ Palaeontology and archaeology
☒	☐ Animals and other organisms
☒	☐ Clinical data
☒	☐ Dual use research of concern
☒	☐ Plants

Methods

n/a	Involved in the study
☒	☐ ChIP-seq
☒	☐ Flow cytometry
☒	☐ MRI-based neuroimaging
